# Cisplatin-activated PAI-1 secretion in the cancer-associated fibroblasts with paracrine effects promoting esophageal squamous cell carcinoma progression and causing chemoresistance

**DOI:** 10.1038/s41419-018-0808-2

**Published:** 2018-07-09

**Authors:** Yun Che, Jingnan Wang, Yuan Li, Zhiliang Lu, Jianbing Huang, Shouguo Sun, Shuangshuang Mao, Yuanyuan Lei, Ruochuan Zang, Nan Sun, Jie He

**Affiliations:** Department of Thoracic Surgery, National Cancer Center/National Clinical Research Center for Cancer/Cancer Hospital, Chinese Academy of Medical Sciences and Peking Union Medical College, Beijing, 100021 China

## Abstract

Preoperative chemotherapy is a promising strategy for the treatment of esophageal squamous cell carcinoma (ESCC). Acquired resistance to chemotherapy is a major obstacle in improving patient prognosis. Cancer-associated fibroblasts (CAFs) are the primary components of the tumor microenvironment and play a crucial role in tumor development; these cells are also potential therapeutic targets for cancer. Using protein arrays, we identified a key secreted cytokine, PAI-1, from CAFs pretreated with cisplatin that was induced after DNA damage of CAFs. The PAI-1 in the tumor microenvironment promoted tumor growth and attenuated the effects of cisplatin treatment. Extracellular PAI-1 activated the AKT and ERK1/2 signaling pathways and inhibited caspase-3 activity and reactive oxygen species accumulation. Tiplaxtinin as a PAI-1 inhibitor could play synergistic effects with cisplatin in vitro and in vivo. In clinical samples, ESCC patients with high expression of PAI-1 in CAFs presented a significantly worse progression-free survival. Taken together, our results showed that PAI-1 secreted from cisplatin-activated CAFs promoted tumor growth and reduced the effects of cisplatin in a paracrine manner, establishing a preclinical rationale to target this cytokine to further improve the clinical response of esophageal squamous cell carcinoma.

## Introduction

Esophageal carcinoma is one of the most common cancers and the leading cause of cancer-related death worldwide^1–4^. Squamous cell carcinoma is the major type of this disease in China, with an estimated 478,000 new cases and 375,000 new deaths in 2015 (ref. ^5^). Despite recent advances in diagnostics and therapeutics, the prognosis for esophageal cancer remains poor, and the 5-year survival rate is approximately 15–25%^1,2^. The standard therapy includes surgery and chemoradiation. Elucidation of the molecular mechanisms of esophageal cancer to help develop new biomarkers and effective therapies is needed. Previous studies of chemoresistance have focused on the tumor cells themselves. However, the host tumor microenvironment (TME) has been completely ignored^6,7^. The TME is comprised of immune cells, fibroblasts, endothelial cells, macrophages, and extracellular matrix (ECM) components, which are believed to play a vital role in inhibiting apoptosis, enabling immune evasion, and promoting proliferation and invasion^8^. Cancer progression and metastasis are known to be controlled by the TME and not solely by cancer cell-autonomous defects. Fibroblasts are a major component of the tumor stroma, and many studies have suggested a prominent functional role for these cells in cancer. Mechanisms of chemoresistance involving the CAFs include the modulation of pathways involving cancer cell-ECM interactions, CAF–ECM adhesion and cytokine- or chemokine-mediated signaling^9^.

Plasminogen activator inhibitor-1 (PAI-1) is a well-known cytokine involved in regulation of vascular fibrinolysis with urokinase-type plasminogen activator (uPA) and its receptor uPAR. PAI-1 is encoded by the SERPINE1 gene. The PAI-1 protein is a serine protease inhibitor (serpin) that functions as the principal inhibitor of tissue plasminogen activator (tPA) and uPA. The inhibition of tPA and uPA resulted in increases in the occurrence and persistence of blood clots^10^. Several reports have examined the function of PAI-1 in cancer, including its role in promoting angiogenesis and preventing apoptosis^11^.

Reactive oxygen species (ROS) have long been associated with cancer and act as a double-edged sword. In cancer, ROS have been shown to induce a variety of biological effects, including DNA damage, cell death, autophagy, and resistance to drugs. Toxic levels of ROS in cancer cells can induce cell apoptosis and senescence. ROS accumulation can affect caspase function^12^. Cisplatin-based chemotherapy is an effective treatment and increases ROS accumulation, resulting in cancer cell apoptosis. The first-line chemotherapy drugs used for esophageal squamous cell carcinoma (ESCC) include cisplatin^13^.

There are many studies showing that CAFs play a vital role in ESCC^14–17^. Nevertheless, the effects of chemotherapy on the CAFs in the TME have not been studied. Here, we hypothesized that drug-treated CAFs could promote ESCC progression and chemoresistance through paracrine effects.

## Methods

### Patients and tumor samples

A total of 49 ESCC tissues were obtained from the Department of Thoracic Surgery of Cancer Hospital of the Chinese Academy of Medical Sciences during Jan 2015 to Jun 2016 in this study (Supplementary Table 1). All patients failed to receive any therapy before operation but received cisplatin-based chemotherapy after surgery. The samples used in the study were approved by the Ethics Committee of Cancer Hospital of the Chinese Academy of Medical Sciences, and all patients provided written informed consent. The clinicopathological characteristics were evaluated and all samples were confirmed by pathological analysis.

### Materials and reagents

RMPI 1640 medium was purchased from HyClone (Logan, UT, USA). Fetal bovine serum (FBS), 100 U/ml penicillin and 100 mg/ml streptomycin were purchased from Gibco (New York, NY, USA). The Cell Counting Kit-8 (CCK-8) reagent was purchased from Dojindo (Kumamoto, Japan). Crystal violet and ROS were purchased from Beyotime (China). All the antibodies (cleaved caspase-3, γH2AX, p-p53, p21, AKT/p-AKT, ERK/p-ERK, and GAPDH) used for the western blot analysis were purchased from Cell Signaling Technologies, Inc. (Danvers, MA, USA) and PAI-1 was purchased from Abcam.

### Cells and cell culture

The human ESCC cell lines KYSE-30 and KYSE-450 and the normal NIH3T3 cells were grown in RMPI 1640 medium (HyClone, Logan, UT, USA). The normal HEK-293 cell lines were maintained in MEM (HyClone, Logan, UT, USA). All culture media were supplemented with 10% FBS (Gibco, New York, NY, USA), 100 U/ml penicillin and 100 mg/ml streptomycin, and all the cell lines were cultured in a 37 °C incubator (Thermo, USA) supplied with 5% CO_2_. Moreover, all the cell lines used in the study were regularly authenticated by short tandem repeat detection and tested for the presence of mycoplasma.

### Isolation and culture of CAFs

Esophageal cancer tissues were obtained immediately from ESCC patients after surgery to acquire stromal fibroblasts. As described previously^18,19^, upon resection, tissue specimens were cut into small pieces of approximately 1 mm^3^, rinsed with phosphate-buffered saline (PBS) and then digested with 1 mg/ml collagenase/dispase (Roche) for 1 h at room temperature in a shaker. After filtration and centrifugation, the cell precipitation was collected and seeded into 10 cm^2^ culture flasks. Thirty minutes later, the medium was replaced with fresh medium to remove non-adherent cells (primarily tumor cells) to obtain pure fibroblasts because the adherence time of fibroblasts (<30 min) is much shorter than that of tumor cells (more than 1 h). After two to three passages, homogeneous CAFs were obtained and prepared for further analysis. The fibroblasts isolated from tumor tissues were defined as CAFs.

### Preparation of the CAF-conditioned medium (CM)

For generation of CM, CAFs were seeded into six-well plates at the indicated cell densities. After adhesion of the cells to the plate overnight, 3 μM cisplatin was added to each well. After 12 h incubation, the medium was replaced with RPMI 1640 medium at a final volume of 2 ml/well with 1% FBS. The CM was collected after 48 h of incubation and stored at – 80 °C.

### Cytokine array

The cytokine secretion of CAFs treated with vehicle control or cisplatin for 12 h was assessed using a 274 human cytokine antibody array from RayBio. This membrane-based cytokine is designed to simultaneously detect the expression levels of multiple cytokines^20^.

### Enzyme-linked immunosorbent assay

Human PAI-1 ELISA kits (Abcam 184863) were used in accordance with the manufacturer’s instructions to quantify the PAI-1 concentration in the culture medium of CAFs^21^.

### Co-culture system

For the transwell co-culture system, KYSE-30 and KYSE-450 cells were plated in 24-well plates and adhered overnight in RPMI 1640 medium. CAFs or cisplatin-pretreated CAFs were plated in the upper chamber with a 0.4 μm pore size (Corning Incorporated, NY, USA) and placed in the top of the same plates. The cells were incubated together for 72 h. Then, the cell viability was calculated by CCK-8 assays.

### Immunofluorescence

A total of 5 × 10^3^ CAFs were seeded in 96-well plates. After overnight adherence, the cells were washed with PBS, fixed with 4% formaldehyde for 15 min, and permeabilized with 0.3% Triton for 10 min. After they were blocked with 5% bovine serum albumin for 30 min, the fixed cells were then incubated with α-SMA (Abcam) or EpCAM (CST) at 4 °C overnight. The next day, cells were washed with PBS and then incubated with anti-mouse Alexa Fluor 488-conjugated secondary antibody (1:1000; Thermo) for 1 h at room temperature. DAPI (Sigma) was then used to counterstain the nuclei. The images were captured using microscopy.

### Apoptosis

Cell apoptosis was assessed using an Annexin V-FITC Apoptosis Detection Kit, according to the manufacturer’s instructions. Briefly, KYSE-30 and KYSE-450 cells pretreated with PAI-1 for 2 h or left untreated were exposed to cisplatin for 24 h. The cells were then collected, washed with binding buffer, and stained with Annexin V-FITC and PI, and then, apoptosis was quantified by flow cytometry (Becton Dickinson FACSCantoII, NJ, USA).

### Plasmid constructs and transfection

For overexpression, the coding DNA sequence of SERPINE1 was cloned into the pLenti-EF1a-EGFP-P2A-Puro-CMV-MCS-3Flag vector. The recombinant plasmid and the packaging plasmid mixture were co-transfected into HEK-293T cells using Lipofectamine 3000 (Invitrogen). The supernatant containing lentivirus was collected and used to infect NIH3T3 cells. The corresponding empty vectors were used as controls. Stable cell lines were selected for 7 days with 1 μg/ml puromycin (Invitrogen). The overexpression efficiencies were controlled by detecting the PAI-1 protein levels via western blot and enzyme-linked immunosorbent assay (ELISA).

### Intracellular ROS measurement

Briefly, the cells were pretreated with PAI-1 for 2 h or left untreated, and then, the indicated concentrations of cisplatin were added for 24 h and incubated with DCFH-DA (10 μmol/l) for 30 min at 37 °C. We washed the cells twice and immediately re-suspended them in PBS to measure ROS levels by flow cytometry.

### Western blotting analysis

Briefly, the cell lysates were separated on SDS-PAGE gels and then transferred to polyvinylidene fluoride (PVDF) membranes (Millipore), which were blocked with 5% milk for 1 h at room temperature. PVDF membranes were incubated with primary antibody overnight at 4 °C, washed, and incubated with secondary antibody for 1 h at room temperature. The immunoreactive bands were visualized according to an ECL western blotting protocol. Antibodies used in the experiment were as follows: cleaved caspase-3, γH2AX, p-ERK1/2, ERK1/2, p-AKT, AKT, PAI-1, and GAPDH.

### In vivo studies

Female BALB/c nude mice (SPF, 4 weeks old) weighing approximately 18 g were purchased from HFK Bioscience Co., Ltd (Beijing, China). The animals were fed a standard commercial diet produced by the Experimental Animal Center of the Chinese Academy of Medical Sciences and were maintained in specific pathogen-free conditions under a 12-h light–dark schedule. For the tissue studies, 1.2 × 10^6^ KYSE-30 cells and CAF _CIS_, NIH3T3^C^, and NIH3T3^PAI-1^ cells were mixed at a 1:1 ratio. The growth of the xenografts was assessed every 3 days. The mice were sacrificed 3 weeks after the tumor injections. For the chemotherapy studies, mice received 2 mg/kg cisplatin and 10 mg/kg tiplaxtinin administered via oral gavage every 3 days for 3 weeks.

### IHC staining

IHC staining of PAI-1 was performed on paraffin-embedded sections of tumor biopsy specimens of ESCC patients. Briefly, sections of 4-μm-thick were dewaxed and rehydrated through graded alcohols. Slides were immunohistochemically stained according to the manufacturer’s instructions. The intensity of PAI-1 expression was graded as 0, negative; 1+, weak cytoplasmic staining; 2+, strong staining in <30% of CAF cells; and 3+, strong staining in more than 30% of CAFs cells. 0 and 1+ were defined as PAI-1 low; and 2+ and 3+ as PAI-1 high. The slides were scored by a pathologist and two experienced researchers independently.

### Statistical analysis

All data were analyzed using GraphPad Prism 6.0 software. The data are expressed as the mean ± SD, and the data from specific experiments were compared by one-way ANOVA or Student’s *t*-test or *Χ*^2^ test. PFS curves were plotted according to the Kaplan–Meier method. *p* < 0.05 was considered statistically significant.

## Results

### Cisplatin-treated CAFs induced proliferation and chemoresistance of ESCC cells in vitro

**Fig. 1:**
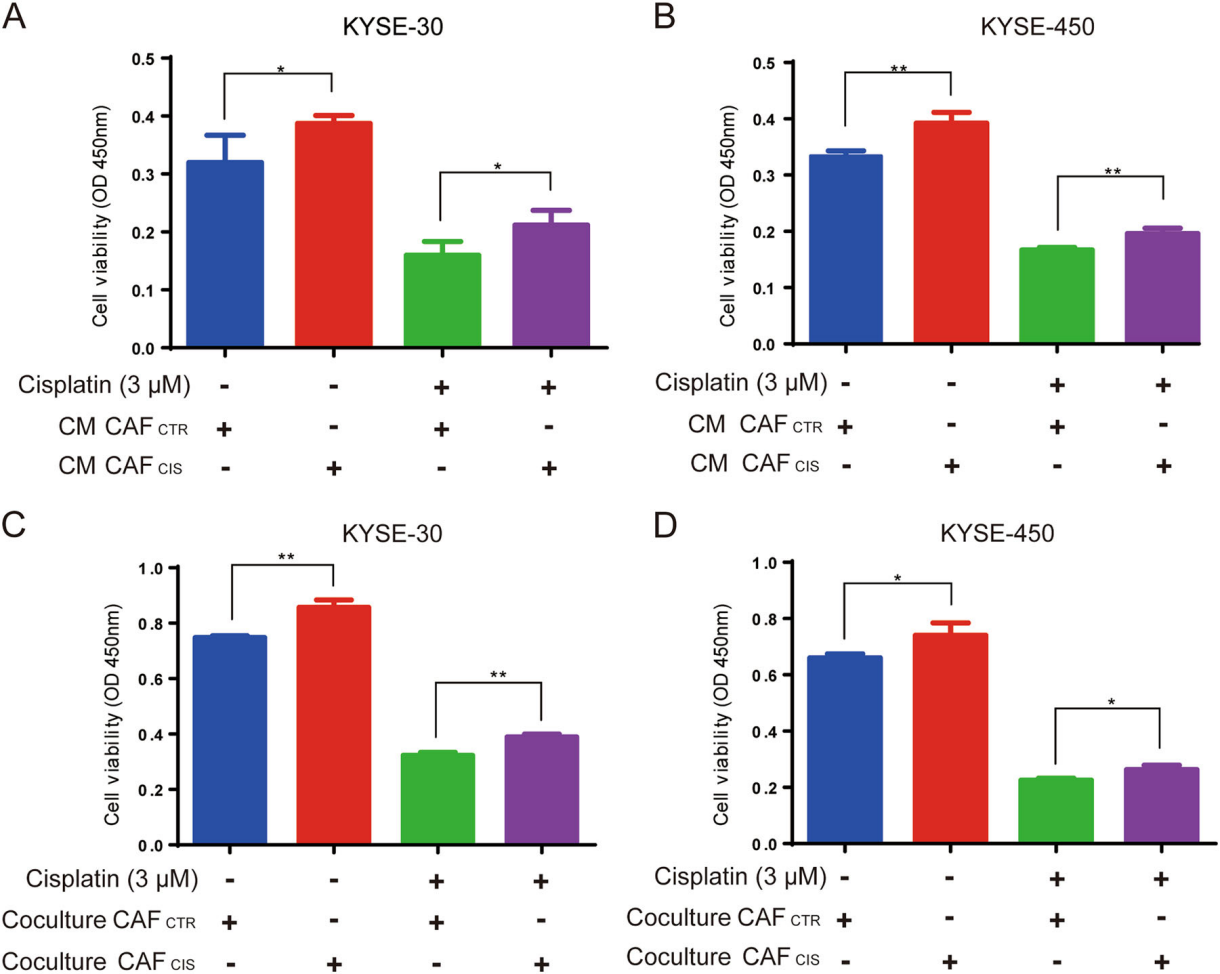
CAFs treated with cisplatin promote ESCC cell growth and cause chemoresistance in vitro. **a**, **b** KYSE-30 and KYSE-450 cells displayed increased proliferation and resistance to cisplatin in CM of CAFs pretreated with cisplatin (CM CAF_CIS_) compared with the control CAFs (CM CAF_CTR_), cell viability was assessed after treatment with 3 μM cisplatin for 72 h using CCK-8 assay. **c**, **d** KYSE-30 and KYSE-450 cells displayed increased proliferation and resistance to cisplatin when co-cultured with CAF_CIS_ compared to CAF_CTR_; cell viability was assessed after treatment with 3 μM cisplatin for 72 h using CCK-8 assays. The results are representative of three independent experiments. *p < 0.05, **p < 0.01 vs the control group

First, CAFs were successfully isolated from two primary ESCC samples by primary culture in RPMI 1640 with 10% FBS as described previously^14^. CAFs showed a spindle-like morphology that was different from the tumor cell morphology. To confirm the characterization of CAFs, we assessed several markers, including α-SMA and EpCAM, that are used to distinguish fibroblasts from tumor cells. The immunofluorescence results showed that the CAFs were stained by the α-SMA antibody but not the EpCAM antibody (Supplementary Figure 1). To determine the effects of treatment-induced damage responses in CAFs on chemoresistance of cancer cells, we co-cultured two ESCC lines, KYSE-30 and KYSE-450, with CM from CAFs treated with cisplatin (CM CAF_CIS_) and without cisplatin (CM CAF_CTR_) for 72 h. The CM CAF_CIS_ promoted the proliferation and chemoresistance of ESCC cells compared with the cells cultured with CM CAF_CTR_ (Fig. 1a, b). A transwell co-culture system of CAFs and cancer cells was established to evaluate the effect of CAFs on proliferation and chemoresistance of ESCC. The pore size of the transwell insert was 0.4 μm. Cells in the co-culture transwell plates were treated with or without cisplatin for 72 h. The viability of ESCC cells was calculated by CCK-8 assays. The results showed that the cell viabilities of KYSE-30 and KYSE-450 cells were strongly increased when the cells were co-cultured with CAFs pretreated by cisplatin (CAF_CIS_) compared with CAFs without any treatment (CAF_CTR_) (Fig. 1c, d). Thus, these data of two co-culture models showed that CAFs pretreated by cisplatin could influence proliferation and chemoresistance of ESCC cells in vitro.

### PAI-1 was the key cytokine secreted by CAFs after cisplatin treatment

**Fig. 2:**
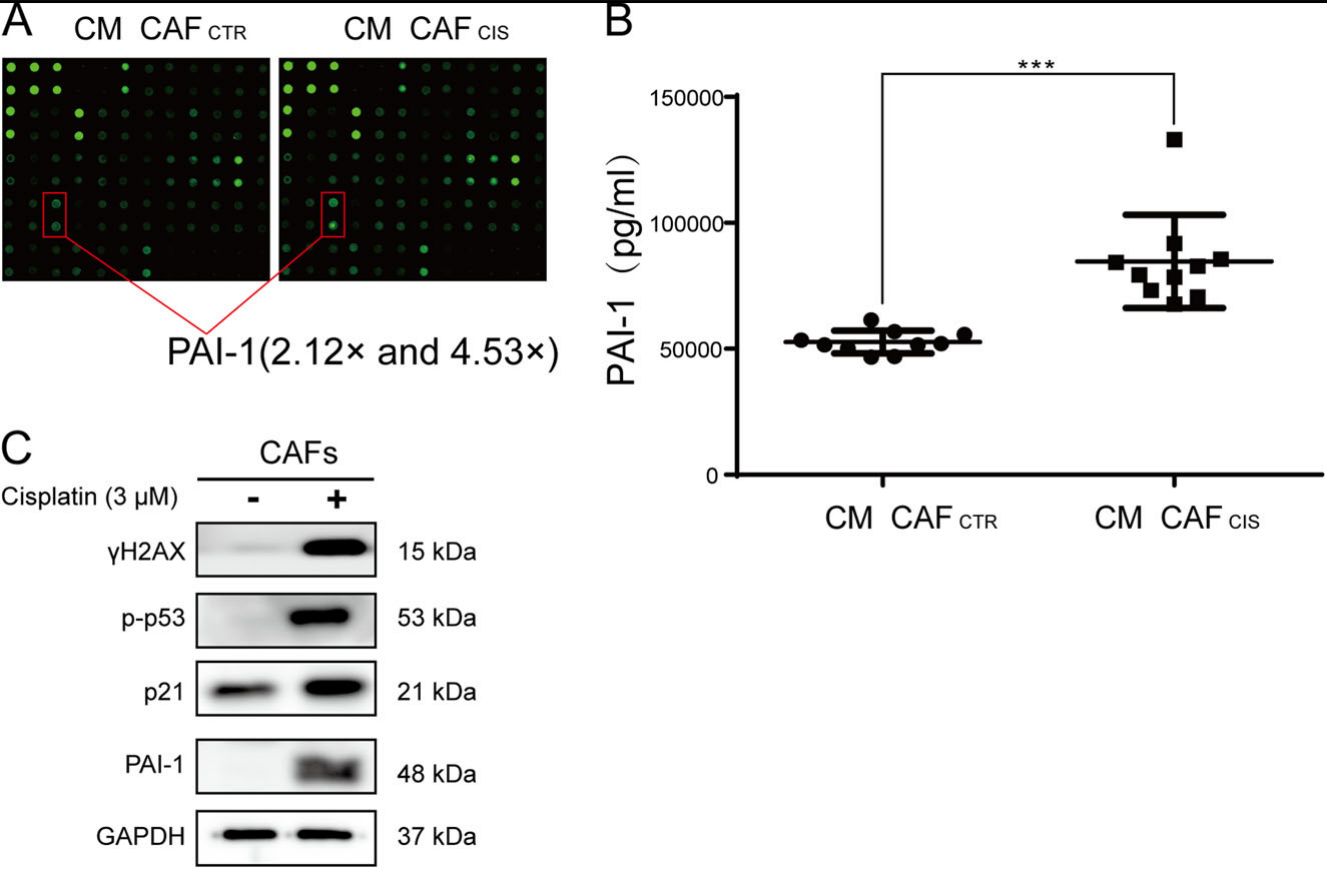
PAI-1 is the key cytokine secreted by CAFs after cisplatin treatment. **a** By using a RayBiotech human cytokine array, we found that the cytokine PAI-1 was upregulated after cisplatin pretreatment of CAFs. **b** By ELISA, the concentration of PAI-1 was confirmed to be higher in the CM CAF_CIS_ compared with the CM CAF_CTR_. **c** Western blot results showed increased expression of γ-H2AX, phosphorylated p53, p21, and PAI-1 in CAFs that received cisplatin treatment compared with cells in the control group. The results are representative of three independent experiments. ***p < 0.001, vs the control group

Next, we determined the differences in cytokines secreted from CAFs treated with cisplatin or without cisplatin. By using the RayBiotech human cytokine array, we found that many cytokines were upregulated or downregulated after cisplatin pretreatment (Supplementary Table 2). Although there were several cytokines unique to each CAFs, there were only five common cytokines in both CAFs groups, the fold change of which were over two times. The results showed that PAI-1 was upregulated over two times in both CAFs, 2.12 and 4.53 times, respectively (Fig. 2a). ELISA (Abcam, ab184863) confirmed that the PAI-1 concentration was much higher in the medium of CM CAF_CIS_ compared with CM CAF_CTR_ (Fig. 2b). However, PAI-1 was far less secreted in KYSE-30 and KYSE-450 cells compared with CAFs (Supplementary Figure 2). The above results suggested the major source of PAI-1 was CAFs but not ESCC cells. Previous studies have reported that PAI-1 was a key regulator of tumor aggressiveness and survival^10,22^. Among the other upregulated cytokines, they did not show obvious effects on ESCC growth and apoptosis, so we chose the cytokine PAI-1 for further study. Cisplatin caused damage to CAFs. CAFs received cisplatin treatment for 24 h, which resulted in a substantial increase in the DNA damage marker γH2AX. Meanwhile, p53 and the p21 cell cycle arrest proteins were phosphorylated (Fig. 2c).

### PAI-1 secreted by CAFs promoted proliferation and colony formation and protected ESCC cells from cisplatin-induced apoptosis

**Fig. 3:**
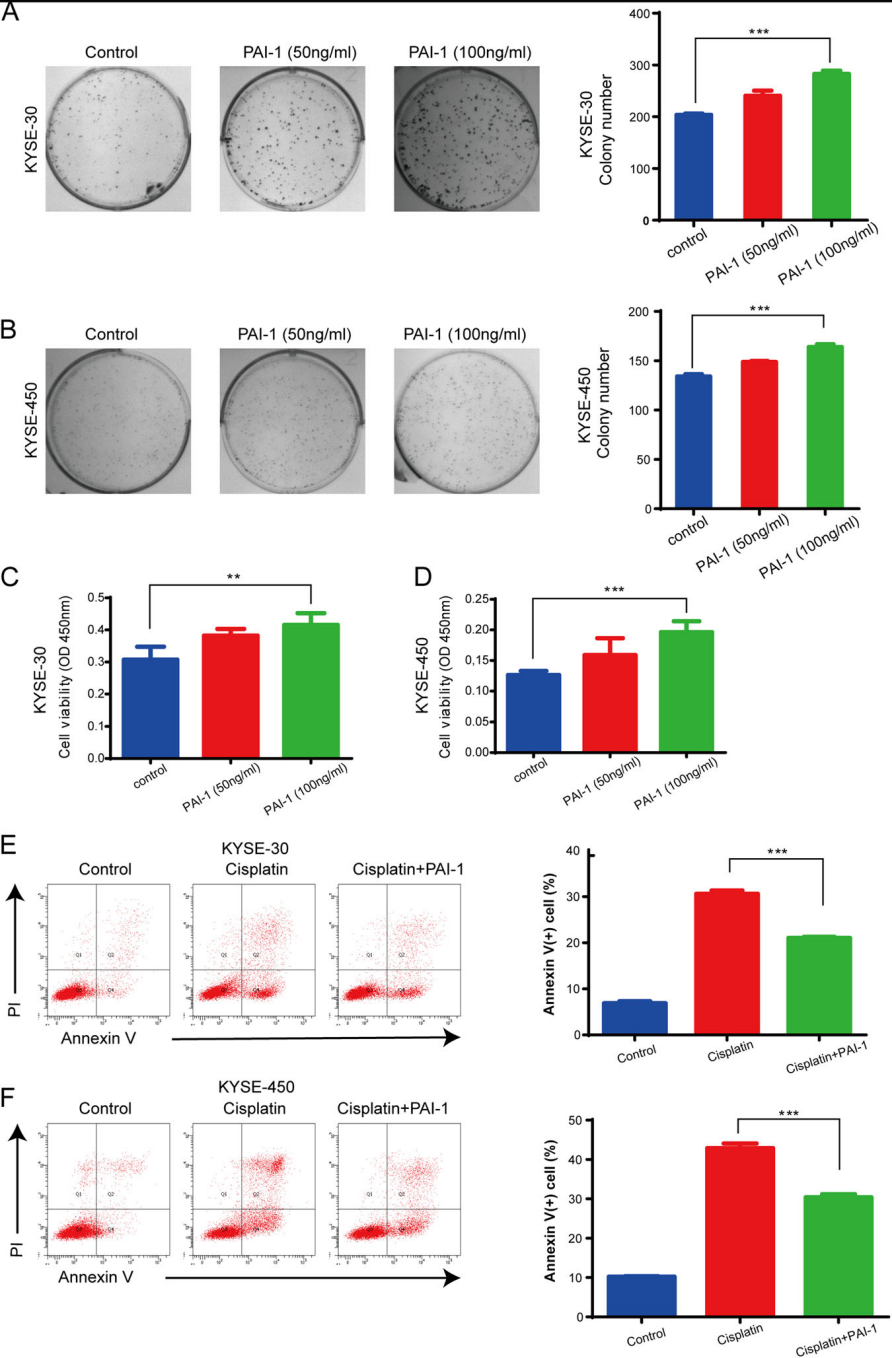
PAI-1 promotes colony formation and cell viability and decreases cisplatin-induced apoptosis of ESCC cells in vitro. **a**, **b** The effect of PAI-1 on proliferation of KYSE-30 and KYSE-450 cells was measured by a colony formation assay. **c**, **d** The effect of PAI-1 on proliferation of KYSE-30 and KYSE-450 cells was measured by CCK-8 assays using different concentrations of PAI-1 (50 and 100 ng/ml). **e**, **f** The effect of PAI-1 on cisplatin-induced apoptosis of KYSE-30 and KYSE-450 cells was analyzed by the Annexin V-FITC Apoptosis Detection Kit. The results are representative of three independent experiments. **p < 0.01, ***p < 0.001, vs the control group

We evaluated the function of PAI-1 from the medium of CAFs pretreated with cisplatin. First, we measured the proliferation of KYSE-30 and KYSE-450 cells by colony formation assays using different concentrations of PAI-1. Colony formation of KYSE-30 and KYSE-450 cells increased substantially in response to PAI-1 compared with that of the control group (Fig. 3a, b). Additionally, we treated KYES-30 and KYSE-450 cells with 50 ng/ml or 100 ng/ml PAI-1 for 72 h. We found that PAI-1 significantly promoted the cell growth rate in a dose-dependent manner (Fig. 3c, d). To validate the importance of PAI-1 in cisplatin-induced apoptosis, KYSE-30 and KYSE-450 cells were treated with cisplatin (3 μM) with or without PAI-1 (100 ng/ml). After 24 h of treatment, cell apoptosis was measured by flow cytometry. In comparison with the cisplatin group, the group treated with PAI-1 showed a decrease in the sensitivity to cisplatin (Fig. 3e, f). When we used tiplaxtinin, a specific inhibitor of PAI-1, together with cisplatin could inhibit the growth of ESCC cells and induce the apoptosis compared with cisplatin alone (Supplementary Figure 3A and B).These results suggest that PAI-1 secreted by CAF_CIS_ enhances survival and promotes chemoresistance of ESCC cells.

### PAI-1 prevented cisplatin-induced DNA damage in ESCC cells through inhibition of ROS accumulation

**Fig. 4:**
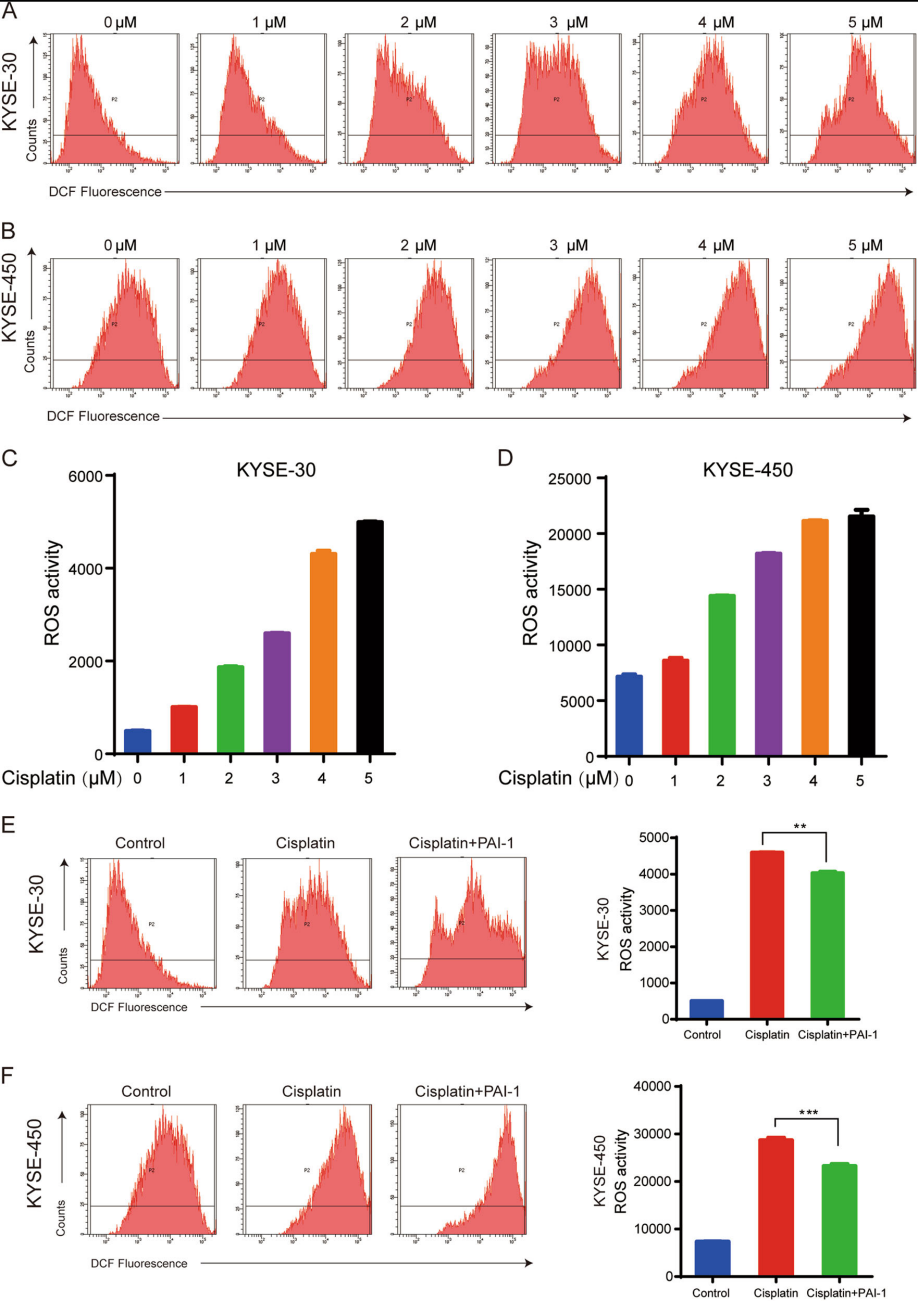
Role of ROS accumulation in chemoresistance of ESCC cells. **a**–**d** ROS levels in KYSE-30 and KYSE-450 cells after 24 h of treatment with different concentrations of cisplatin. **e**, **f** PAI-1 pretreatment 2 h before cisplatin could inhibit ROS accumulation. The data are representative of experiments repeated three times, with similar results. The data are presented as the mean ± S.D. **p < 0.01, ***p < 0.001, vs the control group

Next, we explored the mechanism of PAI-1-mediated chemoresistance. A variety of chemotherapy drugs can disturb the mitochondrial respiratory chain and generate ROS that increase oxidative stress and cell death.^23^ ROS generation plays a critical role in the therapeutic effects of cisplatin. To examine the effect of PAI-1 on the ROS induced by cisplatin in the tumor cells, we detected the total ROS levels in ESCC cells using DCFH-DA. Cisplatin treatment for 24 h significantly increased the intracellular ROS levels as the concentration of cisplatin increased (Fig. 4a–d). However, pretreatment of cells with PAI-1 for 2 h partly inhibited the accumulation of ROS in KYSE-30 and KYSE-450 cells (Fig. 4e, f). Using Tiplaxtinin combined with cisplatin could increase the ROS accumulation of ESCC cells compared with cisplatin alone (Supplementary Figure 3C). Altogether, the data indicate that PAI-1 protects cancer cells against oxidative stress by modulating cellular ROS levels produced by cisplatin.

### Secreted extracellular PAI-1 increased chemoresistance of ESCC cells through inhibition of caspase-3 and γH2AX and activation of AKT and ERK1/2

**Fig. 5:**
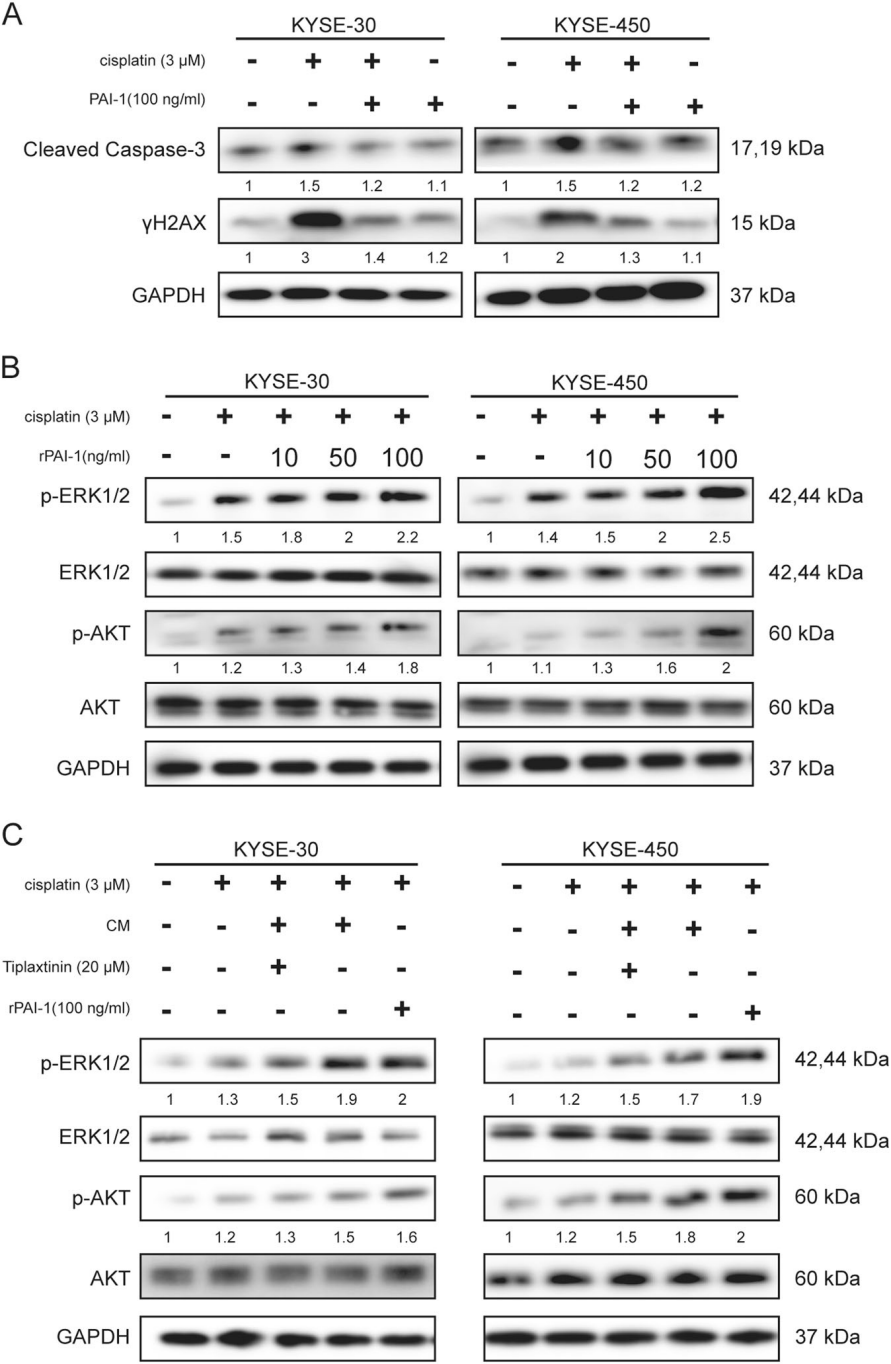
Secreted extracellular PAI-1 increases chemoresistance of ESCC cells by activating the AKT and ERK signaling pathways and inhibiting caspase-3 and γH2AX. **a** The effects of PAI-1 on western blot results of cleaved caspase-3 and γH2AX in KYSE-30 and KYSE-450 cells. **b** Effects of PAI-1 on activation of AKT and ERK1/2 signaling in KYSE-30 and KYSE-450 cells using different concentrations of PAI-1. **c** The effects of CM, PAI-1, and a PAI-1 inhibitor on ERK1/2 and AKT phosphorylation were evaluated by western blot. GAPDH was used as an internal control

Cisplatin induced DNA damage and apoptosis of ESCC cells, which was confirmed by western blot results. Pretreatment with PAI-1 blocked cisplatin-induced phosphorylation of DNA damage and apoptosis markers, such as γH2AX and Cleaved Caspase-3 (Fig. 5a). These results showed that PAI-1 may prevent cisplatin-induced DNA damage and apoptosis.

PAI-1 has been reported as an important cytokine in cell proliferation and chemoresistance that influences several signaling pathways, such as AKT and extracellular signal-regulated kinase (ERK)^22^. After activation of AKT, ERK1/2 is associated with cell survival and death and determines chemoresistance. Thus, we investigated whether PAI-1 could lead to AKT and ERK1/2 activation in KYSE-30 and KYSE-450 cells. As shown in Fig. 5B, phosphorylation of AKT and ERK1/2 in KYSE-30 and KYSE-450 cells was increased by PAI-1 treatment. Tiplaxtinin, a small-molecule inhibitor of PAI-1, effectively inhibited PAI-1 activity. We found that phosphorylation of AKT and ERK1/2 after cisplatin and CM treatment was decreased by tiplaxtinin (Fig. 5c). As shown in Fig. 5c, the cisplatin-induced increase of phosphorylated AKT and ERK1/2 was enhanced by treatment with CM or PAI-1.

Previous studies have reported several ways for paracrine PAI-1 functions on cancer cells. First, PAI-1 can bind to uPA, which binds to uPAR. On this occasion, PAI-1 binds to low-density lipoprotein receptor related protein 1 (LRP-1) and endocytosed in a clathrin-dependent manner^24^. Second, PAI-1 binds to vitronectin to modulate fibrinolysis and cell migration^25^. Third, PAI-1 can inactivate caspase-3 and promote anti-apoptotic effect, while uPAR and LRP-1 are recycled to the cell surface^11^. To investigate how PAI-1 mediates signaling of ESCC cells. KYSE-30 and KYSE-450 cells were treated with two types of inhibitors, tiplaxtinin for PAI-1 inhibition and pitstop-2 to block formation of endocytosis pits. When ESCC cells were treated with tiplaxtinin or pitstop-2, the endocytosis of ESCC cells by PAI-1 was blocked. The expression of intracellular PAI-1 in ESCC cells after PAI-1 treatment was confirmed by western blot (Supplementary Figure 4A). Also, treatment with pitstop-2 did not reduce the phosphorylation of AKT and ERK1/2 obviously by adding PAI-1, while tiplaxtinin could reduce AKT and ERK1/2 phosphorylation (Supplementary Figure 4B). Taken together, these results showed that PAI-1 functioned in enhancing tumor malignancy and chemoresistance through complex formation with its partners, not through clathrin-mediated endocytosis.

### Paracrine PAI-1 facilitated tumor growth and attenuated the effect of cisplatin in vivo

**Fig. 6:**
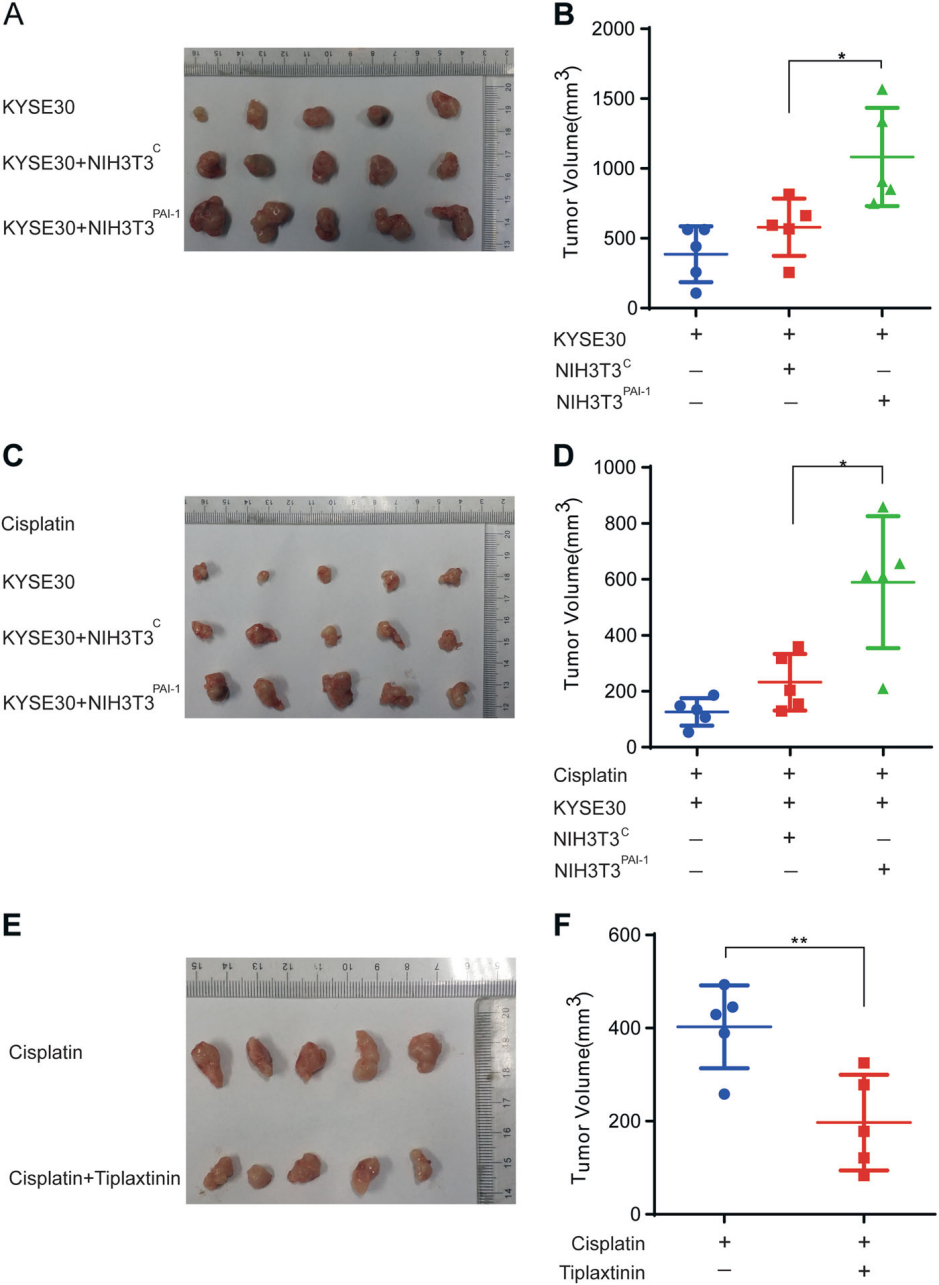
Paracrine PAI-1 induces chemoresistance of ESCC cells in vivo. **a**, **b** NIH3T3 engineered to express PAI-1 promoted the growth of ESCC in vivo. NIH3T3 cells with stable expression of vector control (NIH3T3^C^) and PAI-1 (NIH3T3^PAI-1^) were generated. Tumors derived from KYSE-30 cells alone, KYSE-30 cells in combination with NIH3T3^C^ control or KYSE-30 cells in combination with NIH3T3^PAI-1^ are shown. **c**, **d** In vivo responses of KYSE-30 tumors to cisplatin chemotherapy. Xenografts were derived from KYSE-30 cells alone or KYSE-30 cells combined with either NIH3T3 cells expressing a control vector (KYSE-30 + NIH3T3^C^) or NIH3T3 cells expressing PAI-1 (KYSE-30 + NIH3T3^PAI-1^). Cisplatin was administered every 3 days for 3 weeks. The xenografts were harvested and tumor volumes were determined. Each data point represents an individual xenograft. Horizontal lines are group means of five tumors. **e**, **f** KYSE-30 cells and CAF_CIS_ were subcutaneously injected into the right flanks of nude mice. The animals were treated with cisplatin and cisplatin + tiplaxtinin. *p < 0.05, **p < 0.01, ***p < 0.001 vs the control group

To investigate the in vivo consequences of PAI-1 expression in the TME, NIH3T3 cells with stable expression of vector control (NIH3T3^C^) and PAI-1 (NIH3T3^PAI-1^) were generated (Supplementary Figure 5). Then, we used KYSE-30 cells alone or combined KYSE-30 cells with NIH3T3^C^ or NIH3T3^PAI-1^ cells to construct xenograft models. After 3 weeks, KYSE-30+ NIH3T3^PAI-1^ tumors were larger than KYSE-30+ NIH3T3^C^ and KYSE-30 alone tumors (Fig. 6a). The preceding experiments suggested that in addition to its tumor-promoting effects, paracrine PAI-1 may influence the tumor response to cisplatin in vivo. To test this hypothesis, we treated mice with tumors derived from KYSE-30 cells plus NIH3T3^C^ or NIH3T3^PAI-1^ cells with 2 mg/kg cisplatin every 3 days for 3 weeks. The results showed that KYSE-30 + NIH3T3^PAI-1^ attenuated the tumor inhibitory effects of cisplatin compared with KYSE-30 and KYSE-30 + NIH3T3^C^ (Fig. 6b). We then investigated whether tiplaxtinin, a specific PAI-1 inhibitor, could be used as a drug^26^. Tiplaxtinin was administered by oral gavage to mice bearing KYSE-30 and CAF_CIS_. The tumors treated with cisplatin + tiplaxtinin markedly reduced compared with the cisplatin only tumors (Fig. 6c). Collectively, our data suggested that tiplaxitinin promise an effective therapeutic strategy in enhancing chemotherapeutic effects. These results demonstrated that PAI-1 played an important role in ESCC cells proliferation and chemoresistance in vivo. Tiplaxtinin has potential as an anti-cancer drug in ESCC.

### Expression of PAI-1 in CAFs is positively correlated with poor survival in ESCC patients

**Fig. 7:**
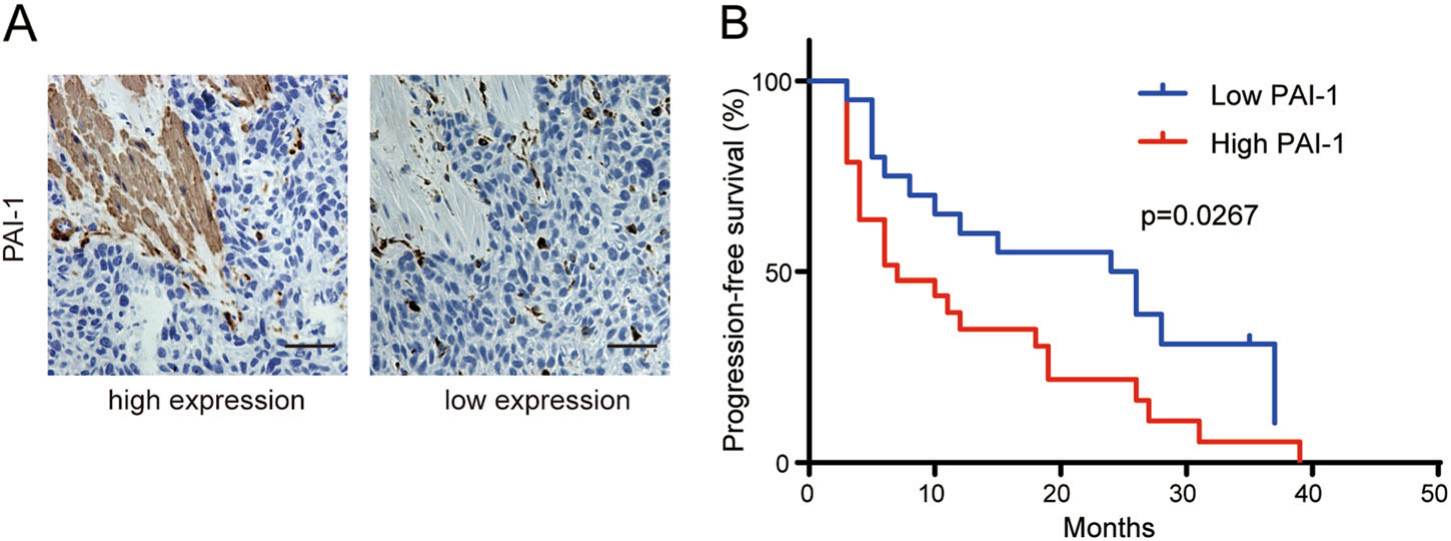
PAI-1 expressed in CAFs is positively correlated with poor survival in ESCC patients. **a** Immunohistochemical staining of PAI-1 expression in CAFs and in tumor tissues in 49 ESCC patients. Scale bar, 50 μm. **b** The Kaplan–Meier curves for PFS of patients who received cisplatin after operation with high and low PAI-1 expression in CAFs

To investigate the clinical significance of PAI-1 in patients’ tumor tissues, we evaluated the correlation between PAI-1 expression in CAFs and clinicopathological characteristics of patients (Supplementary Table 1) and found that patients with high expression of PAI-1 in CAFs presented a significantly worse progression-free survival (PFS) after cisplatin chemotherapy (Fig. 7a, b). These results suggest that high expression of PAI-1 in CAFs is closely correlated with poor PFS in ESCC patients.

## Discussion

**Fig. 8:**
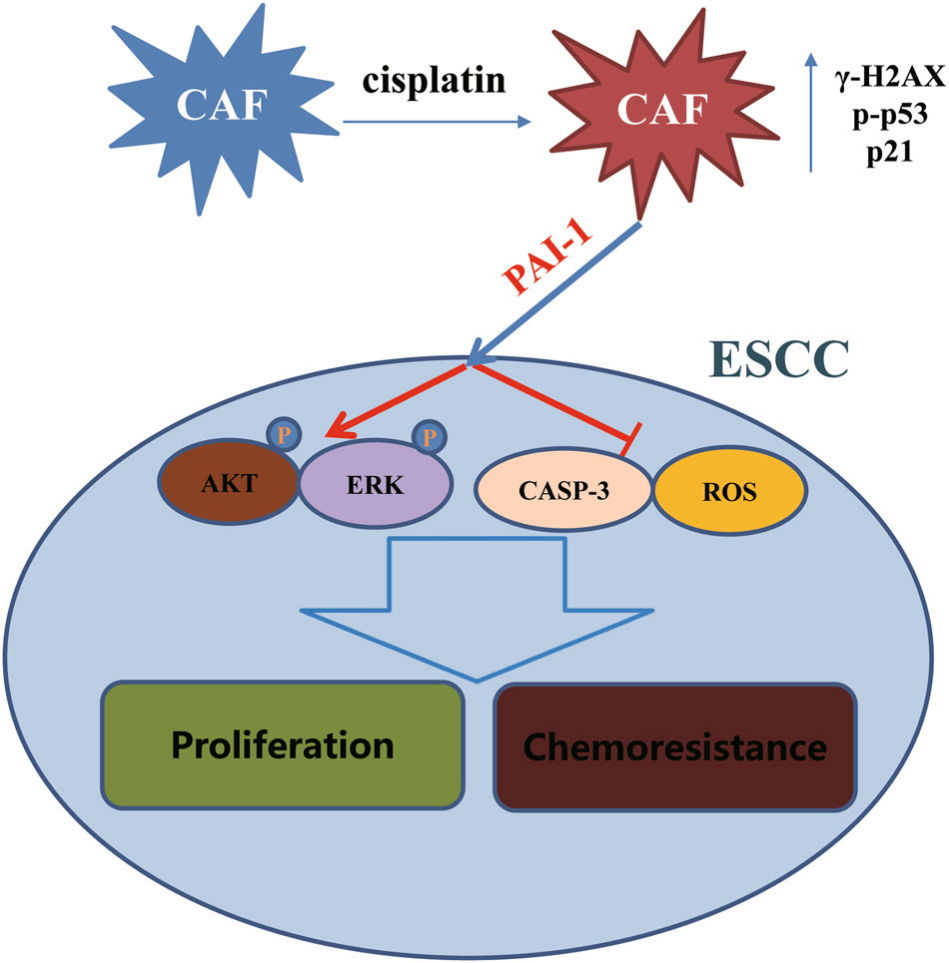
Schematic diagram showing the role of CAFs in mediating ESCC resistance to cisplatin. PAI-1 secreted from CAFs induced by cisplatin activated the AKT and ERK1/2 signaling pathways and inhibited caspase-3 activity and reactive oxygen species accumulation

While a variety of studies have found that cell-autonomous functions play a vital role in mediating acquired resistance to chemotherapy, little is known about the role of tumor stroma in the response to chemotherapy^27^. Cisplatin-based chemotherapy has been widely used to treat many types of cancer, including ESCC^1,2^. Unfortunately, acquired resistance to cisplatin often results in treatment failure and tumor relapse. There are many possible mechanisms of resistance to cisplatin, including increased DNA repair, altered cellular accumulation, and increased drug inactivation^28^. Several reports have found that the TME could modulate the chemotherapy response of cancer cells through paracrine mechanisms. CAFs, a major component of the TME, have been investigated in various cancer processes. However, little is known about the origins and functions of CAFs. Traditionally, CAFs in the TME are primarily distinguished based on their morphology and specific markers, such as α-SMA. CAFs may cause chemoresistance by means of soluble factors^9,29^. Wang et al.^30^ reported that fibroblasts in the TME could cause drug resistance in ovarian cancer cells. Another study reported that CAFs can enhance drug resistance in prostate cancer cells by inhibiting drug accumulation and oxidative stress^31^. Various studies compared the effects of CAFs with those of normal fibroblasts. Additionally, different types of fibroblasts could protect cancer cells from apoptosis. In our research, we investigated the impact of cisplatin on fibroblasts. DNA-damaging drugs not only influence cancer cells but also affect other cellular components in the TME. DNA damage to TME cells can result in the activation of secretory proteins, which affects the growth and resistance of tumor cells^32^. Sun et al.^33^ demonstrated that treatment-induced damage to the TME promotes prostate cancer therapy resistance through WNT16B. The results showed that chemotherapy can cause chemoresistance through cell-nonautonomous effects that are mediated by the fibroblasts in the TME. Another study reported that IL-11 secreted by cisplatin-treated CAFs played a vital role in chemoresistance of lung adenocarcinoma patients^21^. In our study, we investigated the potential cytokines in the CM of pretreated ESCC CAFs. CAFs were isolated from ESCC tissues and were confirmed with immunofluorescence analyses of specific markers. The results of CCK-8 assays demonstrated that CAFs promoted the growth and induced chemoresistance of KYSE-30 and KYSE-450 cells in the co-culture system, and the CM from CAFs had the same effects. Furthermore, we used a protein array to assess the potential cytokines secreted by CAFs. Among all the upregulated cytokines, PAI-1 was upregulated in both samples, and the results were confirmed by ELISA (Fig. 8).

PAI-1 is a protease inhibitor but was paradoxically associated with poor outcomes in cancer patients in a previous study^11^. Many studies have shown that PAI-1 is involved in various cancer processes. The influence of PAI-1 on tumor progression depends on many aspects, such as the concentration, location, type of tumor, and presence of integrins. PAI-1 is known to play a vital role in the pathogenesis of cancer, as it inhibits proteolysis, blocks apoptosis, increases tumor cell adhesion, binds with vitronectin and interacts with integrins, promotes tumor cell proliferation, and affects angiogenesis^34^. Moreover, this protein was correlated with prognosis in esophageal cancer^35–37^. A previous study demonstrated that uPA secreted by CAFs induces ESCC progression^17^. Another study reported that PAI-1 secreted from radioresistant NSCLC cells reduced the radiosensitivity of the nearby cells in a paracrine manner, indicating that functional inhibition of PAI-1 signaling has therapeutic potential^22^. These data strongly indicate that PAI-1 may significantly influence tumor biology, including metastasis and resistance, in a paracrine manner. However, the exact molecular mechanism of PAI-1 is still unclear. We investigated the mechanism of PAI-1 secreted by pretreated CAFs in CAF-mediated chemoresistance^21^. The mechanisms underlying increased PAI-1 secretion may be related to DNA damage-induced p53 expression and p21 expression^38^. In our results, we found that cisplatin increased the DNA damage marker γH2AX, p-p53 and p21. Next, we found that PAI-1 as a paracrine factor could influence proliferation and chemoresistance in ESCC cells in vitro through AKT/ERK activation. Addition of PAI-1 inactivated caspases and γH2AX and decreased the DNA damage of ESCC cells.

Chemotherapy can increase production of ROS to toxic levels, causing programmed cell death, which is known to result in irreparable damage and cell death^13^. Cisplatin, one of the most common chemotherapy drugs used for esophageal cancer, can cause ROS generation (Fig. 4a–d). PAI-1 decreased ROS levels, indicating that PAI-1 inhibits oxidative stress to protect tumor cells against cisplatin (Fig. 4e, f). All the above results demonstrated that PAI-1 inhibits cisplatin-induced tumor cell apoptosis by influencing the ROS-mediated DNA damage^23^.

Finally, we confirmed these results in vivo and in patients’ samples. All the results showed that PAI-1 has the potential to promote tumor growth and reverse cisplatin-induced tumor apoptosis. We found that the worse PFS of ESCC patients was related to the high expression of PAI-1 in CAFs. Tiplaxtinin as a PAI-1 inhibitor combined with cisplatin could inhibit the tumor growth, induce the apoptosis, and increase ROS accumulation in ESCC cells (Supplementary Figure 3). Elucidation of the role of PAI-1 in chemoresistance will be helpful in designing drugs for cancer therapy. Different mechanisms are likely involved because resistance to cisplatin is multilayered and multifactorial. In precision medicine, we not only need to screen the patients before treatment but also during the process of treatment, which will increase the efficacy and decrease the toxicity of platinum-based chemotherapy. Although tiplaxtinin was unsuccessful in clinical trails due to an unfavorable risk to benefit ratio; however, it is still widely used in scientific research. The study described here provided proof for further evaluation of tiplaxtinin combination with chemotherapy.

Taken together, these results describe a model in which chemoresistance results from a cyclical process through cell-nonautonomous effects that are contributed by the TME, primarily CAFs. Our results support the emerging paradigm that chemotherapy-activated CAFs contribute to tumor progression and that targeting the paracrine signaling mediated by activated CAFs may improve the therapeutic effects in ESCC.

## Electronic supplementary material

Supplementary information

Supplementary Figure 1

Supplementary Figure 2

Supplementary Figure 3

Supplementary Figure 4

Supplementary Figure 5
