# Supplementary material for: Cisplatin-activated PAI-1 secretion in the cancer-associated fibroblasts with paracrine effects promoting esophageal squamous cell carcinoma progression and causing chemoresistance
Source: Cell Death Dis. 2018 Jul 9;9(7):759. doi: 10.1038/s41419-018-0808-2 (PMC6037765; doi:10.1038/s41419-018-0808-2)
Supplement: Supplementary file 1 — Supplementary information [file 41419_2018_808_MOESM1_ESM.docx]

**Supplementary information**

Cisplatin-activated PAI-1 secretion in the cancer-associated fibroblasts with paracrine effects promoting esophageal squamous cell carcinoma progression and causing chemoresistance

Yun Che, Jingnan Wang, Yuan Li, Zhiliang Lu, Jianbing Huang, Shouguo Sun, Shuangshuang Mao, Yuanyuan Lei, Ruochuan Zang, Nan Sun*, Jie He*

**Supplementary Figure Legends**

Supplementary Figure 1. Characterization of CAFs. Immunofluorescence was used to distinguish CAFs and tumor cells with antibodies targeting α-SMA (A) and EpCAM (B).

Supplementary Figure 2. The concentration of PAI-1 was confirmed by using ELISA in KYSE-30 CM _CTR_, KYSE-30 CM _CIS_ (A), KYSE-450 CM _CTR_, and KYSE-450 CM _CTR_ (B).

Supplementary Figure 3. Tiplaxtinin combined with cisplatin could inhibit the cell growth (A), induce the apoptosis (B) and increase ROS accumulation (C) of KYSE-30 and KYSE-450 cells compared with cisplatin alone.

Supplementary Figure 4. Secreted PAI-1 stimulated downstream signaling in ECSS cells by extracellular interaction, not by clathrin-mediated endocytosis. (A) Intracellular levels of PAI-1 in KYSE-30 and KYSE-450 cells after treatment with PAI-1，tiplaxtinin and pitstop-2 were confirmed by Western blot. (B) Effects of inhibition of PAI-1 endocytosis on AKT and ERK1/2 phosphorylation in KYSE-30 and KYSE-450 cells were analyzed by using tiplaxtinin or pitstop-2. GAPDH was used as an internal control.

Supplementary Figure 5. The verification data of PAI-1 expression of NIH3T3 cells. (A) The Western blot results of NIH3T3 cells with stable expression of vector control (NIH3T3^C^) and PAI-1 (NIH3T3^PAI-1^). (B) The ELISA results of NIH3T3 cells with stable expression of vector control (NIH3T3^C^) and PAI-1 (NIH3T3^PAI-1^).

**Supplementary Table 1. Association between clinicopathological factors and PAI-1 expression in CAFs in ESCC patients**

|  | Number | PAI-1(+) | PAI-1(-) | P-value |
| --- | --- | --- | --- | --- |
| Gender |  |  |  |  |
| Male | 47 | 28 | 19 | 0.787 |
| Female | 2 | 1 | 1 |  |
|  |  |  |  |  |
| Age (years) |  |  |  |  |
| <60 | 33 | 18 | 13 | 0.834 |
| ≥60 | 16 | 11 | 7 |  |
|  |  |  |  |  |
| Primary Tumor (T) |  |  |  |  |
| T1-2 | 16 | 12 | 4 | 0.117 |
| T3-4 | 33 | 17 | 16 |  |
|  |  |  |  |  |
| Regional Lymph node (N) | | | | |
| N0 | 10 | 5 | 5 | 0.508 |
| N1-3 | 39 | 24 | 15 |  |

| **Supplementary Table 2. All cytokines secretion of CAFs treated with cisplatin and vehicle were assessed using RayBio® Cytokine Antibody Arrays G4000.**  (Abbreviation: A1-CAF1 _CTR_, A2-CAF1 _CIS_, B1-CAF2 _CTR_, B2-CAF2 _CIS_**)** |
| --- |

|  |  | A1 | A2 | B1 | B2 | A2/A1 | B2/B1 |
| --- | --- | --- | --- | --- | --- | --- | --- |
| 6-1 | Angiogenin | 237494.25 | 175007.27 | 266600.48 | 265876.33 | 0.736891 | 0.997284 |
| 6-2 | BDNF | 2495.75 | 4567.94 | 5526.74 | 10192.86 | 1.830288 | 1.844279 |
| 6-3 | BLC | 628.50 | 673.16 | 570.33 | 686.63 | 1.071053 | 1.203928 |
| 6-4 | BMP-4 | 765.50 | 870.73 | 1103.51 | 1017.04 | 1.137463 | 0.92164 |
| 6-5 | BMP-6 | 2085.00 | 1040.63 | 1554.61 | 1149.20 | 0.499101 | 0.739222 |
| 6-6 | CK b 8-1 | 330.75 | 364.89 | 593.33 | 247.55 | 1.103234 | 0.417226 |
| 6-7 | CNTF | 5336.50 | 3989.07 | 3889.35 | 4233.19 | 0.747506 | 1.088406 |
| 6-8 | EGF | 600.50 | 336.90 | 301.79 | 452.84 | 0.561033 | 1.500488 |
| 6-9 | CCL11 | 8767.50 | 5456.37 | 17126.85 | 5111.02 | 0.62234 | 0.298421 |
| 6-10 | CCL24 | 729.00 | 667.04 | 781.19 | 655.10 | 0.915011 | 0.838592 |
| 6-11 | CCL26 | 7919.50 | 5447.04 | 4276.05 | 4850.05 | 0.6878 | 1.134236 |
| 6-12 | FGF-6 | 533.25 | 832.76 | 759.97 | 567.89 | 1.561665 | 0.747258 |
| 6-13 | FGF-7 | 11134.00 | 11767.70 | 26846.50 | 22111.19 | 1.056915 | 0.823615 |
| 6-14 | Flt-3 Ligand | 5528.25 | 3217.12 | 2756.83 | 2954.17 | 0.581942 | 1.071585 |
| 6-15 | CX3CL1 | 545.75 | 639.05 | 691.68 | 604.12 | 1.170955 | 0.873403 |
| 6-16 | GCP-2 | 515.00 | 759.71 | 4810.65 | 7841.46 | 1.475174 | 1.630022 |
| 6-17 | GDNF | 748.75 | 962.76 | 1213.89 | 1479.94 | 1.285818 | 1.219165 |
| 6-18 | CSF2 | 3707.25 | 2041.99 | 2369.41 | 2184.69 | 0.550811 | 0.922037 |
| 6-19 | I-309 | 648.50 | 1057.36 | 580.24 | 1013.68 | 1.630468 | 1.747022 |
| 6-20 | IFN-gamma | 6409.50 | 4634.87 | 5369.30 | 5427.00 | 0.723125 | 1.010746 |
| 6-21 | IGFBP-1 | 9113.50 | 5879.50 | 64441.86 | 54733.90 | 0.645142 | 0.849353 |
| 6-22 | IGFBP-2 | 11902.50 | 8687.65 | 9764.94 | 10352.19 | 0.729901 | 1.060139 |
| 6-23 | IGFBP-4 | 742.25 | 915.78 | 996.31 | 1594.66 | 1.233784 | 1.600568 |
| 6-24 | IGF-I | 622.50 | 628.11 | 715.39 | 561.52 | 1.009009 | 0.784914 |
| 6-25 | IL-10 | 3854.50 | 3245.76 | 3226.67 | 3324.16 | 0.842071 | 1.030212 |
| 6-26 | IL-13 | 10191.75 | 9047.72 | 7812.66 | 7508.04 | 0.887749 | 0.961009 |
| 6-27 | IL-15 | 10316.75 | 8759.41 | 7397.65 | 5736.61 | 0.849047 | 0.775463 |
| 6-28 | IL-16 | 602.75 | 679.59 | 704.07 | 634.31 | 1.127486 | 0.90092 |
| 6-29 | IL-1alpha | 5872.50 | 6222.52 | 3607.37 | 4040.31 | 1.059603 | 1.120017 |
| 6-30 | IL-1beta | 733.50 | 779.34 | 890.52 | 991.21 | 1.062499 | 1.113069 |
| 6-31 | IL-1ra | 4582.00 | 3732.61 | 3656.90 | 3826.64 | 0.814625 | 1.046417 |
| 6-32 | IL-2 | 7868.75 | 3424.35 | 3316.54 | 3519.05 | 0.435183 | 1.061059 |
| 6-33 | IL-3 | 519.50 | 574.05 | 601.82 | 448.14 | 1.105004 | 0.744646 |
| 6-34 | IL-4 | 770.00 | 690.53 | 830.73 | 813.43 | 0.896796 | 0.979177 |
| 6-35 | IL-5 | 6373.75 | 3544.69 | 3057.91 | 3864.21 | 0.556139 | 1.263676 |
| 6-36 | IL-6 | 96450.00 | 143468.64 | 100276.36 | 110101.76 | 1.487492 | 1.097983 |
| 6-37 | IL-7 | 4647.25 | 3563.03 | 4823.03 | 5422.97 | 0.766697 | 1.124391 |
| 6-38 | Leptin | 991.25 | 1352.75 | 1037.35 | 1080.44 | 1.36469 | 1.041536 |
| 6-39 | LIGHT | 729.50 | 1316.71 | 1011.17 | 1075.40 | 1.804949 | 1.063528 |
| 6-40 | MCP-1 | 52587.00 | 61039.75 | 68177.31 | 64136.13 | 1.160738 | 0.940726 |
| 6-41 | MCP-2 | 18566.25 | 15919.26 | 74635.61 | 26413.47 | 0.85743 | 0.353899 |
| 6-42 | MCP-3 | 87945.00 | 292896.59 | 340915.19 | 449290.04 | 3.330452 | 1.317894 |
| 6-43 | MCP-4 | 1078.00 | 1474.38 | 9028.68 | 2317.52 | 1.3677 | 0.256684 |
| 6-44 | M-CSF | 784.50 | 1753.36 | 1574.07 | 1464.51 | 2.235005 | 0.930399 |
| 6-45 | MDC | 845.75 | 1067.01 | 1151.27 | 2008.25 | 1.261616 | 1.744373 |
| 6-46 | MIG | 7286.00 | 4929.94 | 4463.57 | 4721.58 | 0.676632 | 1.057804 |
| 6-47 | MIP-1delta | 422.00 | 544.12 | 473.39 | 495.10 | 1.289394 | 1.045871 |
| 6-48 | MIP-3alpha | 422.50 | 464.65 | 1611.92 | 25570.19 | 1.099753 | 15.86317 |
| 6-49 | NAP-2 | 893.50 | 3192.67 | 869.29 | 1148.86 | 3.573216 | 1.321609 |
| 6-50 | NT-3 | 1504.75 | 1699.30 | 2114.32 | 2296.39 | 1.129292 | 1.08611 |
| 6-51 | PARC | 4840.00 | 6843.55 | 4704.86 | 5398.49 | 1.413956 | 1.147428 |
| 6-52 | PDGF-BB | 883.50 | 1160.97 | 1044.42 | 1462.16 | 1.314058 | 1.399968 |
| 6-53 | RANTES | 1807.25 | 4548.64 | 3307.70 | 5891.91 | 2.516882 | 1.781274 |
| 6-54 | SCF | 1240.75 | 1805.49 | 1550.01 | 1865.35 | 1.455159 | 1.203448 |
| 6-55 | SDF-1 | 1373.00 | 1905.56 | 1812.88 | 2077.35 | 1.387882 | 1.145881 |
| 6-56 | TARC | 578.50 | 824.39 | 735.20 | 776.53 | 1.425051 | 1.056217 |
| 6-57 | TGF-beta1 | 2931.00 | 2525.63 | 2883.49 | 2532.87 | 0.861694 | 0.878404 |
| 6-58 | TGF-beta 3 | 515.50 | 787.39 | 907.86 | 835.23 | 1.527425 | 0.920005 |
| 6-59 | TNF-alpha | 4198.00 | 2789.80 | 3278.33 | 2804.91 | 0.664555 | 0.85559 |
| 6-60 | TNF-beta | 6301.00 | 2800.42 | 3080.91 | 4240.90 | 0.444441 | 1.37651 |
| 7-1 | Acrp30 | 60.50 | 1.06 | 1.11 | 1.15 | 0.017528 | 1.032004 |
| 7-2 | AgRP | 886.25 | 378.84 | 1.11 | 1.15 | 0.42746 | 1.032004 |
| 7-3 | Angiopoietin-2 | 267.25 | 1.06 | 1.11 | 1.15 | 0.003968 | 1.032004 |
| 7-4 | Amphiregulin | 329.50 | 1635.44 | 55753.64 | 55750.25 | 4.963393 | 0.999939 |
| 7-5 | Axl | 1417.00 | 4049.23 | 1838.73 | 3144.04 | 2.857606 | 1.709892 |
| 7-6 | bFGF | 115.50 | 1.06 | 1065.95 | 5615.38 | 0.009181 | 5.267942 |
| 7-7 | b-NGF | 2079.50 | 958.09 | 1.11 | 1.15 | 0.460732 | 1.032004 |
| 7-8 | BTC | 795.00 | 319.19 | 1.11 | 1.15 | 0.401494 | 1.032004 |
| 7-9 | CCL-28 | 874.25 | 315.48 | 175.01 | 1.15 | 0.360853 | 0.006573 |
| 7-10 | CTACK | 871.75 | 483.82 | 1.11 | 1.15 | 0.554997 | 1.032004 |
| 7-11 | Dtk | 1.00 | 1.06 | 1.11 | 1.15 | 1.060423 | 1.032004 |
| 7-12 | EGF-R | 1068.00 | 516.43 | 1.11 | 1.15 | 0.483545 | 1.032004 |
| 7-13 | ENA-78 | 166.50 | 1.06 | 9923.25 | 1318.93 | 0.006369 | 0.132913 |
| 7-14 | Fas/TNFRSF6 | 743.75 | 1411.42 | 1.11 | 1.15 | 1.897713 | 1.032004 |
| 7-15 | FGF-4 | 53.00 | 1.06 | 1.11 | 1.15 | 0.020008 | 1.032004 |
| 7-16 | FGF-9 | 230.50 | 1.06 | 1.11 | 1.15 | 0.004601 | 1.032004 |
| 7-17 | CSF3 | 3910.50 | 2377.47 | 8699.29 | 6656.20 | 0.607971 | 0.765143 |
| 7-18 | GITR-Ligand | 534.50 | 1.06 | 1.11 | 1.15 | 0.001984 | 1.032004 |
| 7-19 | GITR | 106.00 | 1.06 | 1.11 | 1.15 | 0.010004 | 1.032004 |
| 7-20 | GRO | 49721.00 | 28816.21 | 41938.89 | 46133.80 | 0.579558 | 1.100024 |
| 7-21 | GRO-alpha | 2985.25 | 1575.52 | 6303.75 | 4051.99 | 0.52777 | 0.64279 |
| 7-22 | HCC-4 | 617.25 | 99.41 | 1.11 | 1.15 | 0.161061 | 1.032004 |
| 7-23 | HGF | 211401.50 | 238995.07 | 684198.83 | 707528.99 | 1.130527 | 1.034098 |
| 7-24 | ICAM-1 | 1.00 | 1.06 | 1.11 | 1.15 | 1.060423 | 1.032004 |
| 7-25 | ICAM-3 | 1.00 | 1.06 | 1.11 | 1.15 | 1.060423 | 1.032004 |
| 7-26 | IGFBP-3 | 12381.75 | 9272.08 | 6316.01 | 2792.30 | 0.74885 | 0.442099 |
| 7-27 | IGFBP-6 | 5110.00 | 1844.87 | 80.82 | 1.15 | 0.361032 | 0.014235 |
| 7-28 | sIGF-IR | 152.75 | 55.14 | 1.11 | 1.15 | 0.360995 | 1.032004 |
| 7-29 | IL-1 R4/ST2 | 839.50 | 495.75 | 1.11 | 1.15 | 0.590528 | 1.032004 |
| 7-30 | IL-1 RI | 607.50 | 176.83 | 1.11 | 1.15 | 0.291071 | 1.032004 |
| 7-31 | IL-11 | 830.50 | 1004.22 | 3429.72 | 3008.29 | 1.209176 | 0.877124 |
| 7-32 | IL-12 p40 | 1.00 | 1.06 | 1.11 | 1.15 | 1.060423 | 1.032004 |
| 7-33 | IL-12 p70 | 1.00 | 1.06 | 1.11 | 1.15 | 1.060423 | 1.032004 |
| 7-34 | IL-17 | 598.75 | 1.06 | 1.11 | 1.15 | 0.001771 | 1.032004 |
| 7-35 | IL-2 Rapha | 458.50 | 500.78 | 1.11 | 1.15 | 1.092225 | 1.032004 |
| 7-36 | sIL-6 R | 1.00 | 1.06 | 1.11 | 1.15 | 1.060423 | 1.032004 |
| 7-37 | IL-8 | 58621.50 | 54563.83 | 65059.05 | 81646.27 | 0.930782 | 1.254956 |
| 7-38 | I-TAC | 1.00 | 1.06 | 1.11 | 1.15 | 1.060423 | 1.032004 |
| 7-39 | XCL1 | 233.75 | 100.74 | 1.11 | 1.15 | 0.430974 | 1.032004 |
| 7-40 | MIF | 20670.50 | 16070.72 | 19801.36 | 23394.19 | 0.777471 | 1.181444 |
| 7-41 | MIP-1alpha | 100.50 | 249.73 | 1.11 | 1.15 | 2.484873 | 1.032004 |
| 7-42 | MIP-1beta | 1.00 | 1.06 | 1.11 | 1.15 | 1.060423 | 1.032004 |
| 7-43 | MIP-3beta | 56.50 | 1.06 | 1.11 | 1.15 | 0.018769 | 1.032004 |
| 7-44 | MSP-alpha | 1.00 | 1.06 | 1.11 | 1.15 | 1.060423 | 1.032004 |
| 7-45 | NT-4 | 10.75 | 1.06 | 1.11 | 1.15 | 0.098644 | 1.032004 |
| 7-46 | Osteoprotegerin | 543970.50 | 472704.19 | 855394.99 | 793251.69 | 0.868989 | 0.927351 |
| 7-47 | Oncostatin M | 640.25 | 691.93 | 1.11 | 1.15 | 1.080713 | 1.032004 |
| 7-48 | PLGF | 843.50 | 458.10 | 1.11 | 839.79 | 0.543098 | 753.3629 |
| 7-49 | sgp130 | 21903.75 | 16613.65 | 42780.79 | 48946.81 | 0.758485 | 1.144131 |
| 7-50 | sTNF RII | 1.00 | 1.06 | 1.11 | 1.15 | 1.060423 | 1.032004 |
| 7-51 | sTNF-RI | 15650.00 | 16118.97 | 21165.22 | 13768.81 | 1.029966 | 0.65054 |
| 7-52 | TECK | 526.00 | 288.44 | 1.11 | 1.15 | 0.548356 | 1.032004 |
| 7-53 | TIMP-1 | 3838.50 | 3069.93 | 3294.56 | 194.13 | 0.799772 | 0.058924 |
| 7-54 | TIMP-2 | 48261.50 | 51123.55 | 53587.45 | 56083.87 | 1.059303 | 1.046586 |
| 7-55 | Thrombopoietin | 1158.50 | 639.97 | 1570.36 | 934.12 | 0.552409 | 0.594844 |
| 7-56 | TRAIL R3 | 804.75 | 2884.88 | 99.21 | 13335.98 | 3.584818 | 134.4214 |
| 7-57 | TRAIL R4 | 51.75 | 1.06 | 1.11 | 1.15 | 0.020491 | 1.032004 |
| 7-58 | uPAR | 20004.25 | 38487.81 | 3856.94 | 7395.62 | 1.923981 | 1.917484 |
| 7-59 | VEGF | 3993.00 | 3861.00 | 3024.24 | 1460.43 | 0.966943 | 0.482908 |
| 7-60 | VEGF-D | 1.00 | 1.06 | 1.11 | 1.15 | 1.060423 | 1.032004 |
| 8-1 | Activin A | 826.50 | 814.92 | 780.12 | 675.27 | 0.985991 | 0.865594 |
| 8-2 | ALCAM | 1267.50 | 1627.20 | 1257.02 | 1422.61 | 1.283787 | 1.13173 |
| 8-3 | B7-1(CD80) | 693.00 | 624.35 | 337.56 | 651.08 | 0.900936 | 1.928782 |
| 8-4 | BMP-5 | 2254.75 | 2598.81 | 2277.56 | 3117.72 | 1.152593 | 1.368889 |
| 8-5 | BMP-7 | 3587.50 | 3276.03 | 3156.30 | 2843.79 | 0.913179 | 0.900988 |
| 8-6 | CTF1 | 2749.25 | 2636.78 | 2230.21 | 2308.84 | 0.959091 | 1.035256 |
| 8-7 | CD14 | 1087.00 | 1008.86 | 652.80 | 586.67 | 0.928113 | 0.898698 |
| 8-8 | CXCL- 16 | 14528.25 | 8980.24 | 12451.01 | 17506.91 | 0.618123 | 1.406063 |
| 8-9 | DR6 (TNFRSF21) | 4992.50 | 4283.69 | 4166.04 | 4381.10 | 0.858024 | 1.051622 |
| 8-10 | Endoglin | 2747.50 | 2497.88 | 2645.79 | 2735.07 | 0.909145 | 1.033747 |
| 8-11 | ErbB3 | 903.50 | 781.04 | 879.72 | 896.53 | 0.864457 | 1.019103 |
| 8-12 | E-Selectin | 2346.50 | 2131.15 | 2451.74 | 2456.11 | 0.908225 | 1.001782 |
| 8-13 | Fas Ligand | 3436.50 | 3431.76 | 3445.78 | 3182.86 | 0.998619 | 0.923698 |
| 8-14 | ICAM-2 | 695.00 | 660.40 | 630.47 | 538.30 | 0.950211 | 0.853799 |
| 8-15 | IGF-II | 1172.50 | 1337.62 | 869.91 | 1061.75 | 1.140824 | 1.220533 |
| 8-16 | IL-1 R II | 1131.75 | 1294.36 | 1294.56 | 1439.13 | 1.143679 | 1.11168 |
| 8-17 | IL-10 Rbeta | 2054.75 | 2003.30 | 2081.79 | 2315.55 | 0.97496 | 1.112284 |
| 8-18 | IL-13 Ralpha2 | 1991.75 | 2068.19 | 1601.21 | 1459.49 | 1.038376 | 0.911491 |
| 8-19 | IL-18 BPalpha | 2867.00 | 2877.58 | 2811.87 | 3066.72 | 1.00369 | 1.090635 |
| 8-20 | IL-18 Rbeta | 1634.50 | 1561.59 | 1404.46 | 1069.17 | 0.955395 | 0.761269 |
| 8-21 | MMP-3 | 9796.25 | 14318.69 | 113125.77 | 135538.42 | 1.46165 | 1.198122 |
| 8-22 | IL-2 Rbeta | 2387.50 | 2259.00 | 2163.73 | 1936.73 | 0.946177 | 0.895086 |
| 8-23 | IL-2 Rgamma | 3516.00 | 2813.17 | 2522.63 | 2613.67 | 0.800106 | 1.036087 |
| 8-24 | IL-21R | 1690.50 | 1739.43 | 2091.11 | 1977.19 | 1.028943 | 0.945521 |
| 8-25 | IL-5 Ralpha | 1840.50 | 1822.34 | 1698.60 | 2062.92 | 0.990133 | 1.214481 |
| 8-26 | IL-9 | 2892.75 | 3016.24 | 3045.90 | 3184.05 | 1.042691 | 1.045356 |
| 8-27 | IP-10 | 2542.00 | 2369.55 | 3013.03 | 4030.05 | 0.932158 | 1.337542 |
| 8-28 | LAP | 60110.50 | 82207.12 | 61788.96 | 88107.10 | 1.3676 | 1.425936 |
| 8-29 | Leptin R | 594.50 | 716.63 | 804.16 | 637.91 | 1.205435 | 0.793264 |
| 8-30 | LIF | 1148.25 | 1041.54 | 1676.77 | 1899.85 | 0.90707 | 1.133042 |
| 8-31 | L-Selectin | 2690.75 | 2635.58 | 2866.57 | 2888.33 | 0.979496 | 1.007588 |
| 8-32 | CSF1R | 2810.00 | 2329.41 | 2920.79 | 2803.08 | 0.828972 | 0.959699 |
| 8-33 | MMP-1 | 23474.50 | 18059.49 | 42416.46 | 44454.40 | 0.769324 | 1.048046 |
| 8-34 | MMP-13 | 643.00 | 547.21 | 899.59 | 731.78 | 0.851021 | 0.813457 |
| 8-35 | MMP-9 | 585.00 | 333.56 | 406.74 | 472.69 | 0.570193 | 1.162131 |
| 8-36 | MPIF-1 | 3509.50 | 3133.76 | 3539.00 | 3280.32 | 0.892936 | 0.926905 |
| 8-37 | NGF R | 1597.50 | 1178.04 | 1326.45 | 1516.72 | 0.73743 | 1.143442 |
| 8-38 | PDGF AA | 1065.00 | 1730.78 | 1476.10 | 1481.76 | 1.625143 | 1.003836 |
| 8-39 | PDGF-AB | 1256.50 | 1443.60 | 1495.23 | 1556.23 | 1.148903 | 1.040795 |
| 8-40 | PDGF Ralpha | 1016.75 | 1075.43 | 1048.50 | 1001.89 | 1.057711 | 0.955542 |
| 8-41 | PDGF Rbeta | 705.00 | 762.77 | 739.64 | 704.72 | 1.081947 | 0.952786 |
| 8-42 | PECAM-1 | 1086.50 | 1203.04 | 820.84 | 1137.18 | 1.107259 | 1.385379 |
| 8-43 | Prolactin | 2300.50 | 2216.70 | 2393.84 | 2071.30 | 0.963574 | 0.865262 |
| 8-44 | SCF R | 2347.00 | 1007.66 | 1200.11 | 1069.41 | 0.429339 | 0.891096 |
| 8-45 | SDF-1beta | 2513.50 | 2404.87 | 2574.40 | 2555.00 | 0.956782 | 0.992466 |
| 8-46 | Siglec-5 | 2772.00 | 2594.00 | 2809.41 | 2288.01 | 0.935788 | 0.814407 |
| 8-47 | TGF-alpha | 1220.75 | 1063.65 | 1323.26 | 1161.84 | 0.87131 | 0.878016 |
| 8-48 | TGF beta2 | 2061.00 | 1837.96 | 2147.54 | 2166.84 | 0.891781 | 1.008989 |
| 8-49 | Tie-1 | 2423.75 | 2126.10 | 2147.54 | 2106.02 | 0.877195 | 0.980667 |
| 8-50 | Tie-2 | 1011.50 | 634.44 | 1080.88 | 1306.71 | 0.627229 | 1.208932 |
| 8-51 | TIMP-4 | 1853.00 | 1908.37 | 1384.10 | 1783.71 | 1.029883 | 1.288716 |
| 8-52 | CDH5 | 2045.00 | 2312.83 | 2687.73 | 2566.50 | 1.130968 | 0.954892 |
| 8-53 | VEGF R2 | 1485.00 | 1876.41 | 1670.88 | 1963.78 | 1.263576 | 1.175299 |
| 8-54 | VEGF R3 | 2277.00 | 2791.79 | 3122.93 | 3017.63 | 1.226081 | 0.966281 |
| 9-1 | Adiposin | 11182.50 | 2235.98 | 18245.15 | 5580.27 | 0.199953 | 0.30585 |
| 9-2 | BCAM | 912.00 | 1098.63 | 1341.24 | 5159.06 | 1.20464 | 3.846479 |
| 9-3 | CD30 | 10593.25 | 7069.23 | 17429.46 | 13794.34 | 0.667334 | 0.791438 |
| 9-4 | CD40 | 1053.50 | 1570.91 | 2632.67 | 9508.91 | 1.491136 | 3.611881 |
| 9-5 | Fcr RIIB/C | 1307.50 | 1613.24 | 2526.64 | 9814.99 | 1.233833 | 3.884605 |
| 9-6 | Ferritin | 1244.75 | 1926.03 | 3223.96 | 17502.10 | 1.547319 | 5.428757 |
| 9-7 | FLRG | 1183.50 | 2002.16 | 1977.97 | 5173.77 | 1.691726 | 2.615692 |
| 9-8 | FSTN | 2971.50 | 3781.85 | 7069.25 | 30169.48 | 1.272709 | 4.267708 |
| 9-9 | Furin | 1292.00 | 1259.93 | 1124.80 | 3661.35 | 0.975178 | 3.255097 |
| 9-10 | LGALS7 | 906.50 | 1156.96 | 2375.67 | 11343.13 | 1.27629 | 4.774705 |
| 9-11 | GDF-15 | 820.50 | 1113.86 | 2148.45 | 10526.06 | 1.357536 | 4.899371 |
| 9-12 | hGH | 217794.50 | 203633.74 | 237002.03 | 228628.60 | 0.934981 | 0.964669 |
| 9-13 | IL-10 Rα | 593.00 | 585.32 | 662.66 | 1533.97 | 0.987045 | 2.314866 |
| 9-14 | IL-22 | 194313.00 | 202721.96 | 255921.65 | 237262.87 | 1.043275 | 0.927092 |
| 9-15 | IL-28A | 40076.50 | 56014.74 | 103866.62 | 79861.42 | 1.397695 | 0.768884 |
| 9-16 | IL29 | 1245.00 | 1678.27 | 2775.68 | 12546.17 | 1.348009 | 4.52003 |
| 9-17 | IL-31 | 21722.25 | 51930.68 | 82066.83 | 71412.01 | 2.390667 | 0.870169 |
| 9-18 | Insulin | 1265.75 | 1526.52 | 2946.93 | 8065.97 | 1.206022 | 2.737075 |
| 9-19 | LH-β | 1512.75 | 1414.00 | 3138.46 | 7614.83 | 0.934722 | 2.426293 |
| 9-20 | LIMPII | 1128.00 | 1234.90 | 2313.80 | 1865.92 | 1.094766 | 0.806434 |
| 9-21 | LYVE-1 | 832.50 | 1212.19 | 2211.61 | 6018.98 | 1.456079 | 2.721539 |
| 9-22 | Marapsin | 2071.50 | 3353.45 | 5165.22 | 20686.95 | 1.618849 | 4.005049 |
| 9-23 | MICA | 2061.00 | 2466.70 | 2907.91 | 8693.10 | 1.196844 | 2.989472 |
| 9-24 | MICB | 2253.00 | 7716.23 | 96740.38 | 68547.19 | 3.424869 | 0.708569 |
| 9-25 | MMP-2 | 4037.50 | 6432.56 | 7130.35 | 26131.55 | 1.593203 | 3.664833 |
| 9-26 | MMP-7 | 16963.00 | 26663.95 | 40035.95 | 54410.00 | 1.571889 | 1.359028 |
| 9-27 | MMP-8 | 660.50 | 404.92 | 609.77 | 716.14 | 0.613054 | 1.174441 |
| 9-28 | MMP-10 | 922.00 | 1631.04 | 1958.97 | 3236.84 | 1.769028 | 1.652314 |
| 9-29 | NCAM-1 | 267.00 | 288.53 | 443.66 | 814.79 | 1.080635 | 1.836526 |
| 9-30 | NID1 | 2100.00 | 2941.56 | 4261.47 | 11181.34 | 1.400741 | 2.623821 |
| 9-31 | NrCAM | 1444.00 | 1843.96 | 2815.99 | 3216.30 | 1.276978 | 1.142154 |
| 9-32 | NRG1-beta1 | 1739.50 | 1971.45 | 3030.37 | 9748.55 | 1.133341 | 3.216946 |
| 9-33 | OPN | 1953.50 | 2902.84 | 3241.68 | 3485.10 | 1.485971 | 1.075093 |
| 9-34 | PAI-I | 2806.75 | 5974.99 | 6461.01 | 29275.44 | 2.128792 | 4.53109 |
| 9-35 | PF4 | 807.00 | 1039.02 | 1584.38 | 5353.31 | 1.287504 | 3.378803 |
| 9-36 | PSA-total | 1305.75 | 1935.06 | 3340.52 | 14473.46 | 1.481951 | 4.332692 |
| 9-37 | RAGE | 2711.25 | 3523.78 | 4011.91 | 15401.09 | 1.299687 | 3.83884 |
| 9-38 | RANK | 2481.25 | 6054.99 | 60478.59 | 56962.65 | 2.440299 | 0.941865 |
| 9-39 | Resistin | 3535.00 | 6443.65 | 5657.66 | 17255.61 | 1.822816 | 3.049957 |
| 9-40 | SAA | 12897.25 | 24962.19 | 42928.97 | 60865.92 | 1.935466 | 1.417829 |
| 9-41 | Siglec-9 | 1175.25 | 1498.65 | 2239.34 | 11827.49 | 1.275176 | 5.281687 |
| 9-42 | TACE | 522.50 | 852.17 | 444.94 | 1772.86 | 1.630945 | 3.984474 |
| 9-43 | KIM1 /TIM1 | 371.00 | 521.06 | 1331.74 | 2088.07 | 1.404465 | 1.567923 |
| 9-44 | TRAIL R2 | 840.50 | 1211.67 | 436.47 | 2267.61 | 1.441605 | 5.195364 |
| 9-45 | Trappin-2 | 370.00 | 455.25 | 1955.38 | 4631.59 | 1.230397 | 2.368641 |
| 9-46 | TREM-1 | 697.25 | 510.99 | 546.87 | 634.48 | 0.732867 | 1.160213 |
| 9-47 | TSH-β | 1072.25 | 1237.74 | 959.46 | 1455.11 | 1.154334 | 1.516588 |
| 9-48 | TSLP | 3293.00 | 6935.03 | 8054.38 | 19728.37 | 2.105992 | 2.449397 |
| 9-49 | VCAM-1 | 623.00 | 1087.53 | 3329.74 | 3816.80 | 1.745641 | 1.146276 |
| 9-50 | VEGF-C | 1228.00 | 2690.19 | 5054.82 | 25490.47 | 2.190708 | 5.042808 |
| 9-51 | XEDAR | 2152.75 | 4068.06 | 7574.01 | 26853.27 | 1.889704 | 3.54545 |
| 10-1 | 4-1BB | 1100.75 | 934.30 | 1018.32 | 1343.48 | 0.848782 | 1.31931 |
| 10-2 | ACE-2 | 2600.50 | 1827.37 | 1744.28 | 1149.94 | 0.7027 | 0.659261 |
| 10-3 | AFP | 3069.75 | 13530.90 | 3549.17 | 11619.49 | 4.407817 | 3.273862 |
| 10-4 | ANGPT 1 | 9209.00 | 8952.71 | 7682.50 | 2500.09 | 0.972169 | 0.325426 |
| 10-5 | Angiostatin | 146.75 | 143.20 | 131.64 | 205.44 | 0.975779 | 1.560568 |
| 10-6 | ANGPTL4 | 2502.75 | 2798.82 | 2376.17 | 2468.17 | 1.118299 | 1.038718 |
| 10-7 | beta 2 M | 743.75 | 915.85 | 925.07 | 1021.10 | 1.231402 | 1.103808 |
| 10-8 | BCMA | 170.00 | 226.73 | 255.06 | 152.05 | 1.333684 | 0.596127 |
| 10-9 | beta IG-H3 | 4303.00 | 3966.08 | 3627.06 | 5114.50 | 0.921702 | 1.410096 |
| 10-10 | CA125 | 583.50 | 741.20 | 867.75 | 771.85 | 1.270265 | 0.889479 |
| 10-11 | CA15-3 | 433.50 | 458.88 | 400.96 | 345.88 | 1.058539 | 0.86262 |
| 10-12 | CA19-9 | 15869.75 | 16909.55 | 18616.06 | 21187.77 | 1.065521 | 1.138145 |
| 10-13 | CA9 | 530.50 | 593.66 | 468.98 | 617.77 | 1.119067 | 1.317256 |
| 10-14 | CTSS | 2409.00 | 2771.16 | 2821.01 | 3547.59 | 1.150336 | 1.25756 |
| 10-15 | CCL14 | 1402.50 | 943.79 | 856.51 | 814.50 | 0.672933 | 0.950958 |
| 10-16 | CCL21 | 885.25 | 918.30 | 880.92 | 1028.36 | 1.037329 | 1.167371 |
| 10-17 | CD23 | 1233.25 | 1029.49 | 13.99 | 148.57 | 0.834777 | 10.62161 |
| 10-18 | CD40 Ligand | 3361.00 | 2593.25 | 115.46 | 156.69 | 0.771571 | 1.35707 |
| 10-19 | CEA | 44.00 | 9.22 | 117.38 | 1.74 | 0.209567 | 0.014832 |
| 10-20 | CEACAM-1 | 192.75 | 214.79 | 264.66 | 177.29 | 1.114362 | 0.669892 |
| 10-21 | Cripto-1 | 3295.50 | 2835.71 | 88.59 | 418.42 | 0.860478 | 4.723386 |
| 10-22 | CRP | 4636.00 | 4530.46 | 5334.04 | 5239.27 | 0.977234 | 0.982234 |
| 10-23 | DAN | 314.75 | 238.93 | 524.93 | 447.44 | 0.759112 | 0.85238 |
| 10-24 | Decorin | 4715.75 | 2755.16 | 277.82 | 978.74 | 0.584246 | 3.522878 |
| 10-25 | DKK-1 | 2667.50 | 3570.94 | 2793.31 | 2910.38 | 1.338684 | 1.041911 |
| 10-26 | DKK-3 | 1115.00 | 1194.65 | 1069.60 | 1336.51 | 1.071437 | 1.24954 |
| 10-27 | DKK-4 | 33.50 | 89.50 | 31.81 | 64.42 | 2.671559 | 2.024817 |
| 10-28 | DPP4 | 404.00 | 371.55 | 420.99 | 439.89 | 0.919676 | 1.044916 |
| 10-29 | CDH1 | 1148.50 | 1263.54 | 1272.56 | 1109.89 | 1.100164 | 0.872176 |
| 10-30 | EDA-A2 | 1016.75 | 1246.18 | 1263.78 | 982.80 | 1.225651 | 0.777667 |
| 10-31 | EG-VEGF | 1399.00 | 1214.99 | 1783.77 | 1770.31 | 0.868472 | 0.992456 |
| 10-32 | EpCAM | 1704.50 | 1737.06 | 1938.45 | 2237.78 | 1.019102 | 1.154413 |
| 10-33 | HER2 / ErbB2 | 1535.25 | 590.14 | 295.65 | 490.96 | 0.384393 | 1.660628 |
| 10-34 | EPO R | 2968.50 | 2343.20 | 2445.56 | 1486.82 | 0.789355 | 0.607969 |
| 10-35 | FSH | 1208.75 | 1225.57 | 1469.75 | 1350.44 | 1.013915 | 0.918826 |
| 10-36 | HB-EGF | 4652.00 | 2466.06 | 1187.54 | 1456.64 | 0.530107 | 1.22661 |
| 10-37 | hCG intact | 1452.50 | 2445.72 | 2324.06 | 2020.15 | 1.683797 | 0.869233 |
| 10-38 | HVEM | 140.50 | 157.84 | 132.19 | 171.20 | 1.12342 | 1.295077 |
| 10-39 | IL-13 R alpha1 | 4005.75 | 5679.55 | 5424.82 | 5468.80 | 1.417849 | 1.008107 |
| 10-40 | IL-17B | 689.50 | 1685.26 | 563.33 | 810.44 | 2.444177 | 1.43867 |
| 10-41 | IL-17C | 539.50 | 540.78 | 637.92 | 521.72 | 1.002373 | 0.817844 |
| 10-42 | IL-17F | 48.50 | 13.02 | 1.10 | 41.20 | 0.268408 | 37.55944 |
| 10-43 | IL-17 R | 2522.25 | 2554.74 | 2334.48 | 2220.95 | 1.012881 | 0.951366 |
| 10-44 | CALCA | 1966.50 | 1748.72 | 2085.46 | 2274.63 | 0.889256 | 1.09071 |
| 10-45 | PSA-free | 2571.50 | 3000.87 | 3836.04 | 3092.90 | 1.166972 | 0.806274 |
| 10-46 | S-100b | 1967.25 | 2105.63 | 2153.47 | 2155.66 | 1.07034 | 1.001015 |
| 10-47 | Shh N | 800.50 | 700.52 | 739.67 | 670.58 | 0.875102 | 0.906587 |
| 10-48 | TG | 8212.50 | 10253.67 | 10288.78 | 10424.57 | 1.248544 | 1.013199 |
| 10-49 | Ubiquitin | 2015.00 | 2241.23 | 2492.45 | 2246.48 | 1.112272 | 0.901313 |
