# Supplementary figures and images for: Cisplatin-activated PAI-1 secretion in the cancer-associated fibroblasts with paracrine effects promoting esophageal squamous cell carcinoma progression and causing chemoresistance
Source: Cell Death Dis. 2018 Jul 9;9(7):759. doi: 10.1038/s41419-018-0808-2 (PMC6037765; doi:10.1038/s41419-018-0808-2)

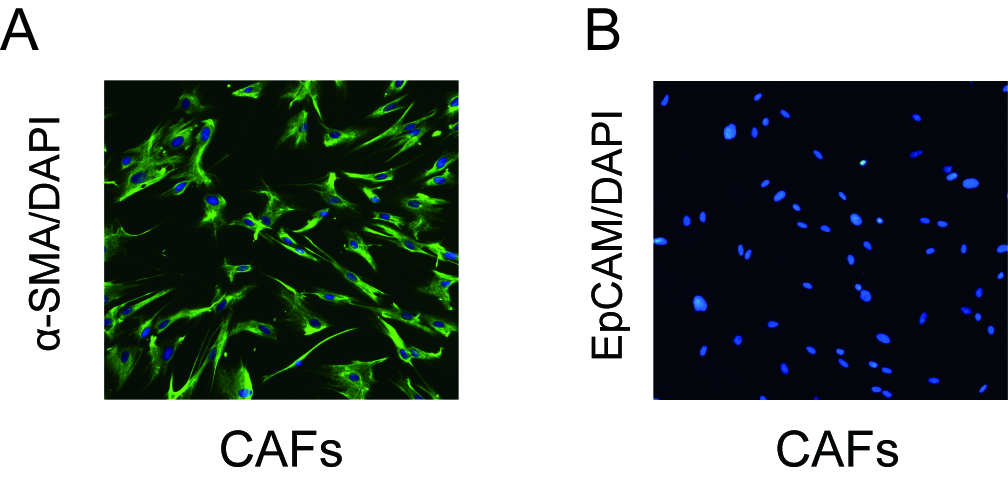

Supplement: Supplementary file 2 — Supplementary Figure 1 [file 41419_2018_808_MOESM2_ESM.tif]

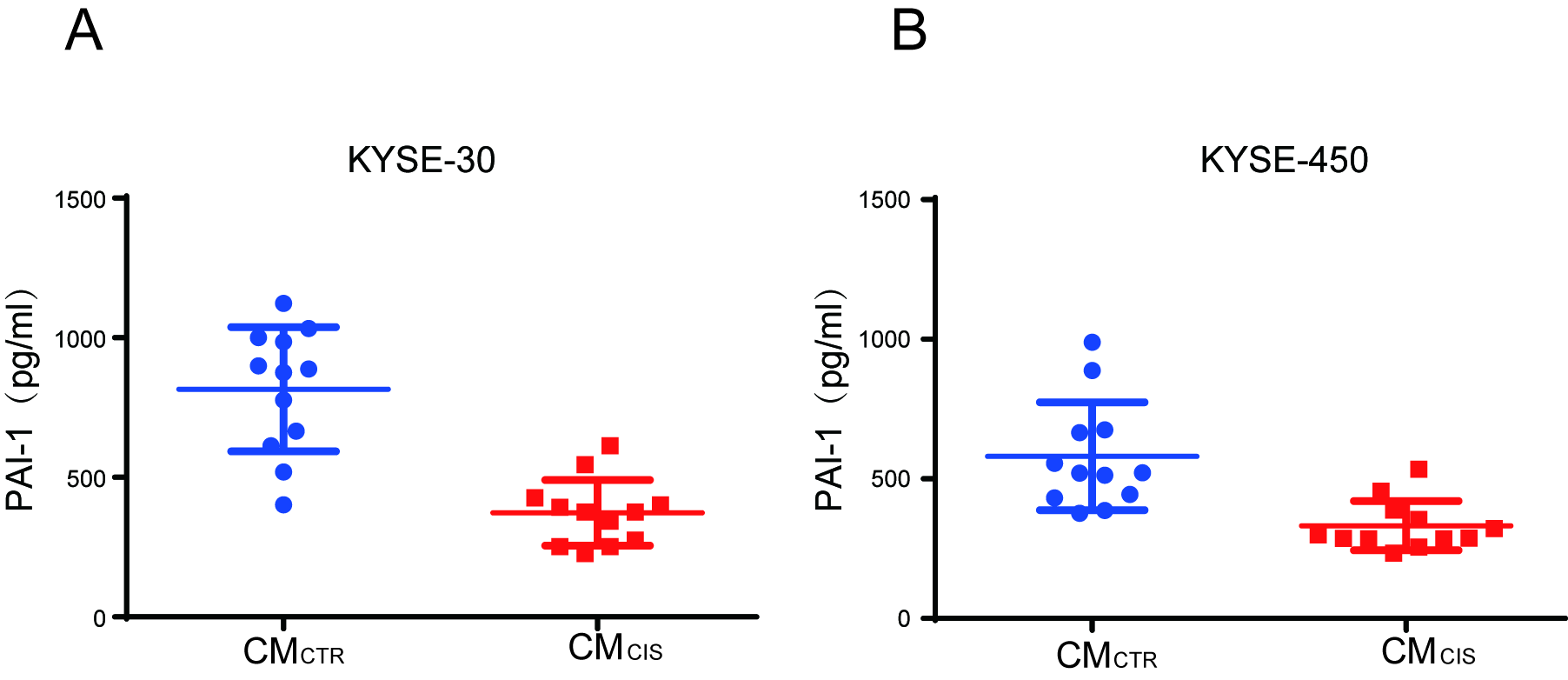

Supplement: Supplementary file 3 — Supplementary Figure 2 [file 41419_2018_808_MOESM3_ESM.tif]

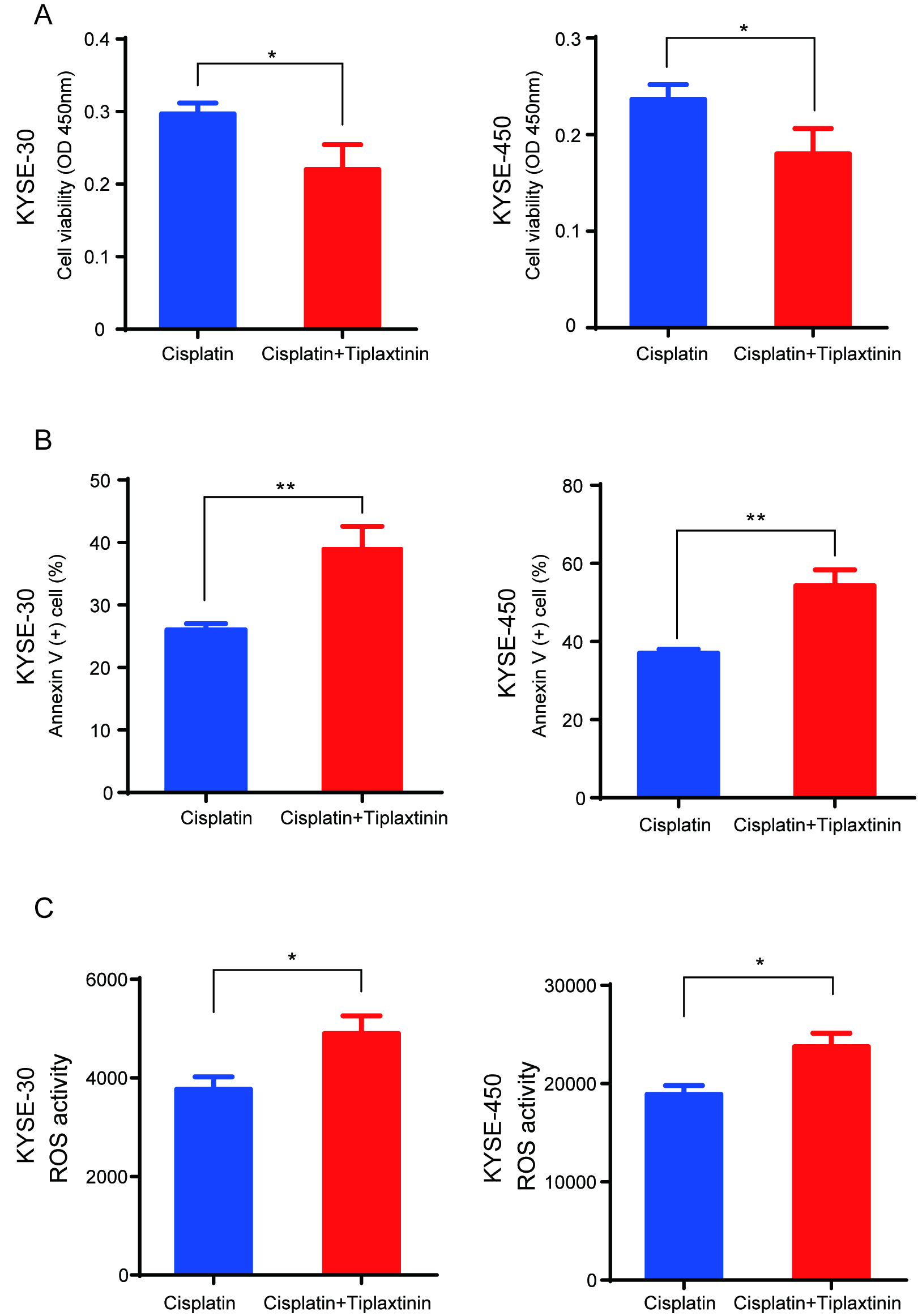

Supplement: Supplementary file 4 — Supplementary Figure 3 [file 41419_2018_808_MOESM4_ESM.tif]

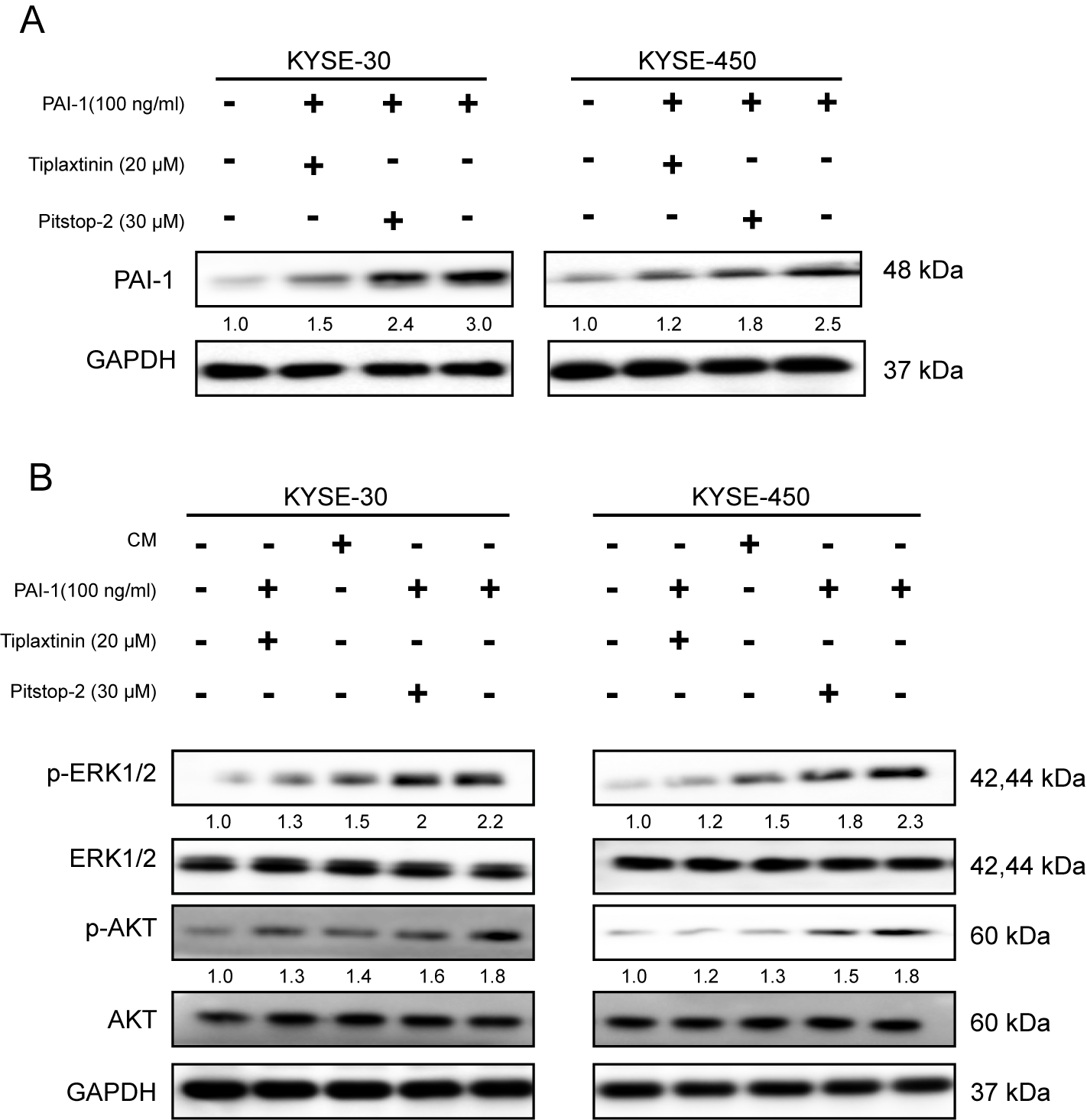

Supplement: Supplementary file 5 — Supplementary Figure 4 [file 41419_2018_808_MOESM5_ESM.tif]

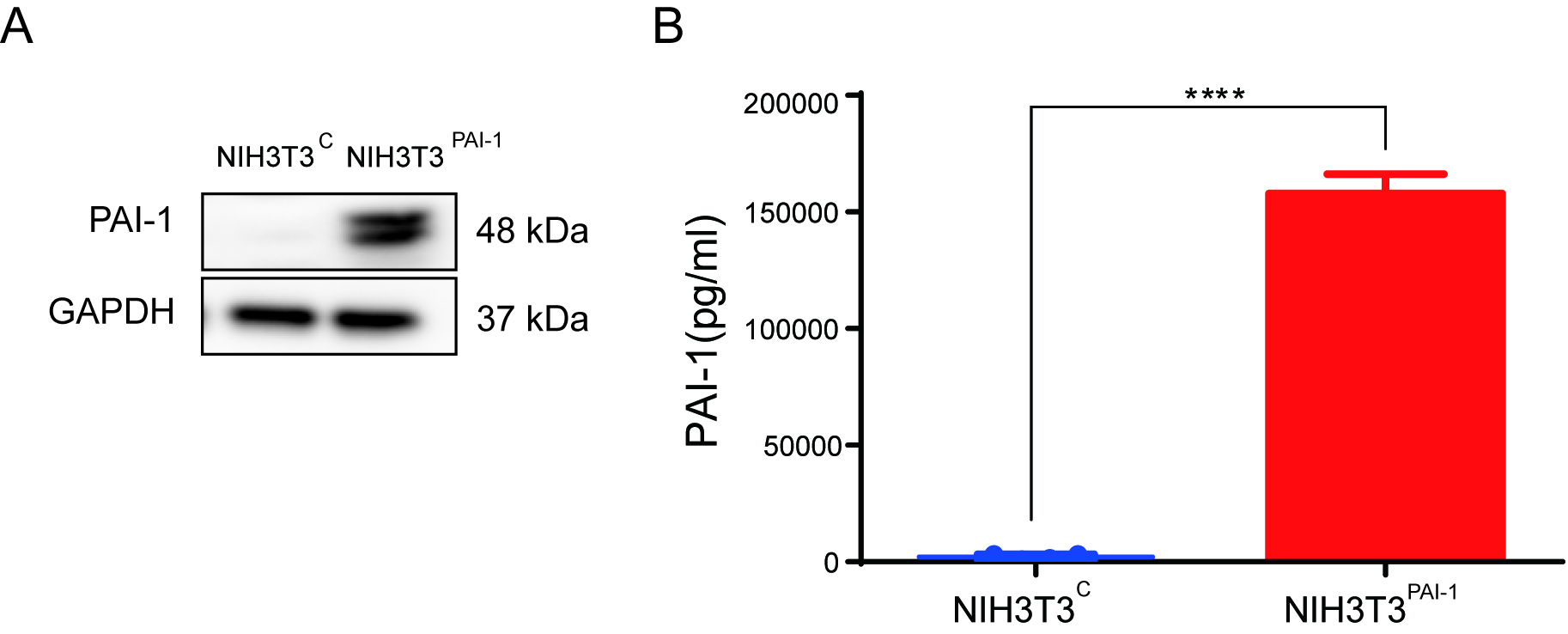

Supplement: Supplementary file 6 — Supplementary Figure 5 [file 41419_2018_808_MOESM6_ESM.tif]
